# A rapid research needs appraisal methodology to identify evidence gaps to inform clinical research priorities in response to outbreaks—results from the Lassa fever pilot

**DOI:** 10.1186/s12916-019-1338-1

**Published:** 2019-06-11

**Authors:** Louise Sigfrid, Catrin Moore, Alex P. Salam, Nicola Maayan, Candyce Hamel, Chantelle Garritty, Vittoria Lutje, Brian Buckley, Karla Soares-Weiser, Rachel Marshall, Mike Clarke, Peter Horby

**Affiliations:** 1 Centre for Tropical Medicine and Global Health, University of Oxford, Oxford, UK; 2 United Kingdom Public Health Rapid Support Team, London, UK; 3 Cochrane Response, Cochrane, London, UK; 4 Knowledge Synthesis Group, Clinical Epidemiology Program, Ottawa Hospital Research Institute, Ottawa, Canada; 5 Cochrane Infectious Diseases Group, Liverpool School of Tropical Medicine, Liverpool, UK; 6 Department of Surgery, Philippine General Hospital, National University of the Philippines, Manila, Philippines; 7 Editorial & Methods Department, Cochrane Central Executive, Cochrane, London, UK; 8 London, UK; 9 Evidence Aid, Centre for Public Health, Queen’s University Belfast, Belfast, UK

**Keywords:** Emerging infectious diseases, Clinical research priorities, Outbreak response, Lassa fever, Rapid research needs appraisal methodology

## Abstract

**Background:**

Infectious disease epidemics are a constant threat, and while we can strengthen preparedness in advance, inevitably, we will sometimes be caught unaware by novel outbreaks. To address the challenge of rapidly identifying clinical research priorities in those circumstances, we developed and piloted a protocol for carrying out a systematic, rapid research needs appraisal (RRNA) of existing evidence within 5 days in response to outbreaks globally, with the aim to inform clinical research prioritization.

**Methods:**

The protocol was derived from rapid review methodologies and optimized through effective use of pre-defined templates and global time zones. It was piloted using a Lassa fever (LF) outbreak scenario. Databases were searched from 1969 to July 2017. Systematic reviewers based in Canada, the UK, and the Philippines screened and extracted data using a systematic review software. The pilot was evaluated through internal analysis and by comparing the research priorities identified from the data, with those identified by an external LF expert panel.

**Results:**

The RRNA pilot was completed within 5 days. To accommodate the high number of articles identified, data extraction was prioritized by study design and year, and the clinical research prioritization done post-day 5. Of 118 potentially eligible articles, 52 met the data extraction criteria, of which 46 were extracted within the 5-day time frame. The RRNA team identified 19 clinical research priorities; the expert panel independently identified 21, of which 11 priorities overlapped. Each method identified a unique set of priorities, showing that combining both methods for clinical research prioritization is more robust than using either method alone.

**Conclusions:**

This pilot study shows that it is feasible to carry out a systematic RRNA within 5 days in response to a (re-) emerging outbreak to identify gaps in existing evidence, as long as sufficient resources are identified, and reviewers are experienced and trained in advance. Use of an online systematic review software and global time zones effectively optimized resources. Another 3 to 5 days are recommended for review of the extracted data and to formulate clinical research priorities. The RRNA can be used for a “Disease X” scenario and should optimally be combined with an expert panel to ensure breadth and depth of coverage of clinical research priorities.

**Electronic supplementary material:**

The online version of this article (10.1186/s12916-019-1338-1) contains supplementary material, which is available to authorized users.

## Background

The need and ability to conduct clinical research during infectious disease outbreaks to inform current and future responses is gaining acceptance as a core pillar of outbreak response [1]. However, the unpredictable nature of outbreaks makes outbreak research challenging [2, 3]. Since time is of the essence, researchers, policy makers, and funders need to rapidly identify key gaps in evidence at the earliest stages of an outbreak, so that they can prioritize research to address those gaps and ensure that any research that is undertaken has maximal value [4]. Traditional approaches to evidence assimilation, such as systematic reviews, require time and resources that are unlikely to be available during an outbreak, with traditional systematic reviews generally taking at least 12 months [5]. Rapid reviews are a variation of a systematic review that balances time constraints with considerations in bias [6], designed to inform healthcare policies and guidelines [7]. Even rapid reviews and scoping reviews might require 1 to 6 months or longer to complete [5, 6, 8, 9]. Furthermore, there is no gold standard approach for rapid or scoping reviews, with methods varying greatly [5–11]. This highlights a need for a robust methodology to rapidly and systematically identify key gaps in knowledge and evidence to inform research prioritization early in outbreaks. To address this, we developed and piloted a transparent and replicable protocol for carrying out a rapid research needs appraisal (RRNA), within 5 days in response to (re-) emerging outbreaks globally. The aim is not to identify and fully appraise all the available evidence, since this is not feasible within 5 days. Rather, the aim is to identify important gaps in evidence and knowledge in a robust, systematic, and replicable manner to rapidly inform clinical research prioritization.

The RRNA was piloted in 2017 using a fictitious Lassa fever (LF) outbreak scenario. LF is an acute, viral illness first recognized in Nigeria in 1969 [12]. The causative pathogen, Lassa virus (LASV), is a zoonotic, single-stranded RNA arenavirus that is endemic in Guinea, Liberia, Sierra Leone, and Nigeria, with seasonal peaks in incidence. A limited number of cases have also been reported from Benin, Burkina Faso, Côte d’ Ivoire, Ghana, and Togo [13]. LASV has been prioritized by the World Health Organization (WHO) as a high threat pathogen for which there is a need for accelerated research and development [14]. In the beginning of 2018, Nigeria experienced a large outbreak of LF, with 1999 suspected cases reported by the Nigeria Centre for Disease Control. Of 437 confirmed cases, 109 died, giving a case fatality rate (CFR) of 25% in confirmed cases [15]. This article presents the development of the RRNA methodology, its piloting and evaluation using LF as an example, and the research priorities that were identified.

## Methods

**Fig. 1:**
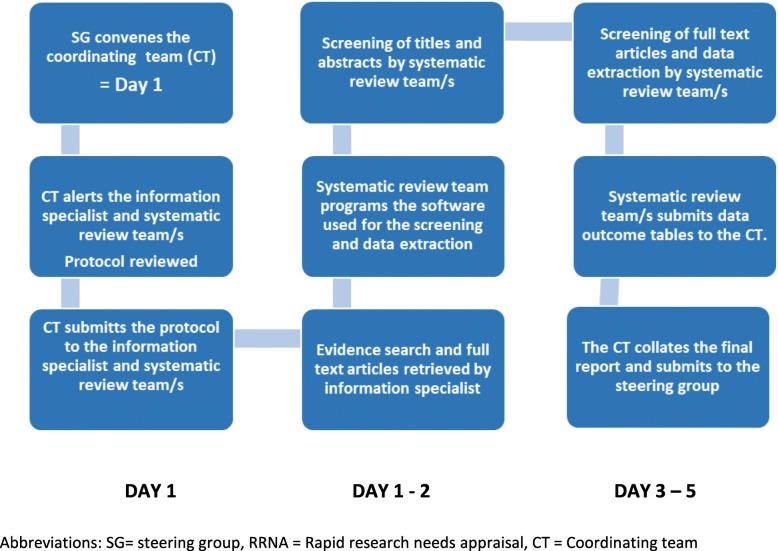
The 5-day rapid research needs appraisal process

The RRNA protocol was developed as a collaboration between Cochrane Response, the Ottawa Hospital Research Institute, Evidence Aid, and the UK Public Health Rapid Support Team staff at the University of Oxford. The methodology was derived from existing rapid review methodologies and optimized through the use of global teams of systematic reviewers across different time zones, pre-defined screening and data extraction templates and the use of an online systematic review software (DistillerSR, Evidence Partners, Ottawa, Canada). The protocol was focused on identifying existing evidence gaps across pre-defined clinical domains (Additional file 1). These were defined by researchers and clinicians with previous experience of clinical research outbreak response. The RRNA protocol is registered on the Open Science Framework [16], and an online training package has been developed [17]. The 5-day process is illustrated in Fig. 1.

### Protocol development

A working group of experts with experience in systematic and rapid review methodologies, information management, and clinical infectious disease research was convened. The protocol was developed over 4 months through three face-to-face meetings with members of the working group and e-mail iterations [16]. The methodology was designed to be used for (re-) emerging infectious diseases where existing evidence and knowledge is expected to be limited and for the purposes of this exercise was focused on clinical aspects, rather than epidemiological, animal, or ecological studies. Therefore, the protocol was designed to be inclusive with a focus on identifying all relevant articles presenting the outcomes of clinical research, including conference abstracts and ongoing clinical trials. The protocol was piloted in a two-stage process. Prior to piloting, experienced systematic reviewers based across three different time zones (Canada, the UK, the Philippines) were engaged and trained in the methodology and the use of the DistillerSR online systematic review software. The systematic reviewers programmed the generic RRNA screening and data extraction forms into DistillerSR prior to piloting. A Dropbox folder was set up for sharing of documents. An online instant messenger group was also set up to allow queries to be posted and answered rapidly by all members of the team.

The processes, including the evidence search strategy, the handover of information, and the data extraction template were initially piloted over 1 day using a fictitious Nipah virus outbreak scenario. This process mini-pilot was evaluated through feedback from all involved in the pilot at a conference meeting call. The feedback informed updates to the data extraction table and the handover processes. The final RRNA protocol was subsequently piloted fully over 5 days using a fictitious LF outbreak scenario.

### Lassa fever pilot

Outside of this pilot study, the process would be triggered when a decision is taken that there is a need to carry out a RRNA in response to a (re-) emerging outbreak. Depending on the setup, this decision may be taken by a steering group, which can be separate or the same as the coordinating team (CT). For this pilot, the steering group based at the University of Oxford, consisting of clinical researchers with experience in infectious disease outbreak response and systematic reviews also acted as the CT. The pilot was triggered by the CT on 17 July 2017. At the start of the pilot, the CT reviewed and updated the search databases and inclusion criteria considering the nature of the outbreak scenario. The updated protocol, specific to the LF outbreak scenario, was then submitted via e-mail to the information specialist based in Glasgow, UK, and the systematic review teams based in London, UK; Ottawa, Canada; and Manila, the Philippines, together with the fictitious outbreak report and supporting clinical information about LF from the US Centers for Disease Control and Prevention (CDC).

**Fig. 2:**
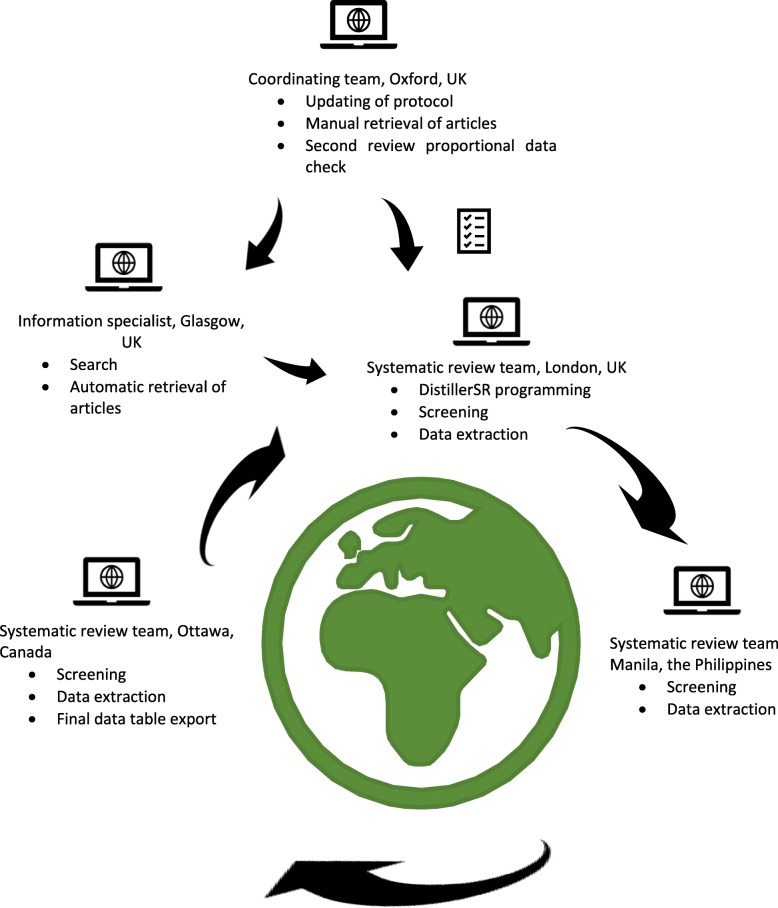
The rapid research needs appraisal pilot global “relay” teams

Figure 2 illustrates the tasks carried out by the systematic review teams involved in the Lassa fever pilot.

The review team based at Cochrane Response in the UK updated the protocol in DistillerSR accordingly, while the information specialist carried out the search. The search was completed on day 1, and the result submitted as an Endnote library to the systematic review team in London for uploading into DistillerSR.

#### Search strategy

An information specialist searched the following electronic databases for articles published from 1969 until 17 July 2017: MEDLINE (PubMed), EMBASE (OVID), the Cochrane Library (including the Cochrane Database of Systematic Reviews and Cochrane Central Register of Controlled Trials), DARE (Database of Abstracts of Reviews of Effect), Epistemonikos, and PROSPERO. Moreover, the following trial registries: Clinicaltrials.gov, the WHO International Clinical Trials Registry Platform (ICTRP), the ISRCTN registry, and the websites of WHO, CDC, and the European Centre for Disease Prevention and Control (ECDC), using the search terms described in Box 1. There were no language restrictions applied.

#### Eligibility

One systematic reviewer screened titles and abstracts for inclusion using DistillerSR, with a second reviewer screening all papers deemed ineligible by the first reviewer. Any disagreements were passed to the next level of screening. Full-text articles were screened independently by two reviewers and consensus on disagreements reached by involving a third reviewer. Articles not found through the automated or manual search were excluded due to non-accessibility given the time constraints. These were listed with full bibliography in the final report. All articles presenting human, clinical LF quantitative research were included. Qualitative studies, non-human animal and cell studies, were excluded. Studies only presenting data on community transmission from zoonotic sources were excluded, since the objective was on clinical research. There were no language limitations at the screening stages.

#### Data extraction

The data extraction was limited to core data essential for identifying gaps across the pre-defined clinical research domains (Additional file 1). The pre-defined data extraction table was designed to facilitate extraction of (1) bibliography, (2) study design, (3) study objectives, (4) number of participants, (5) populations covered, (6) interventions and/or exposure, (7) comparators, and (8) outcomes [16]. Risk of bias assessment or grading of evidence beyond the above data extraction was not done.

The protocol specified that one systematic reviewer performed the data extraction from the included papers. A second researcher would then do a random check of 10% of all qualitative data and 100% of the quantitative data extracted. For prioritization purposes, articles would be extracted in order of study design and publication date, with study designs providing a higher level of evidence and most recently published articles prioritized. Data would not be extracted from studies whose design provided a lower level of evidence, such as case series and case reports, when data were available from studies providing a higher level of evidence for the clinical domains addressed in the article. The final data extraction table was submitted to the CT as an Excel file by mid-day on day 5, together with the list of bibliographies of the articles not extracted, with reasons provided and a PRISMA flow chart.

#### Gaps analysis and clinical research prioritization

The final data extraction table was reviewed by one member of the CT, who organized and summarized the level of evidence and outcomes identified for each clinical domain and incorporated the findings into a final report post-day 5 of the RRNA process. The final report was submitted to the CT. The members of the CT subsequently identified gaps in evidence by reviewing the extracted data for each clinical domain, by study design, study objectives, populations covered, interventions/exposure, and outcomes presented and through a round of iterations and consensus discussions over 2 days defined key clinical research priorities, informed by the gaps in evidence identified.

#### Pilot evaluation

The full RRNA pilot was evaluated in a two-step process. Firstly, a brief survey was sent to everyone involved in the pilot, asking about facilitators and barriers experienced. The survey responses were collated by the CT and discussed during a telephone conference a week after completion of the pilot. Secondly, an expert panel of three LF experts was asked to independently identify seminal articles and LF clinical research priorities. The panel members independently identified clinical research priorities and seminal articles from their previous experience of Lassa fever research. These were submitted to the team at Oxford University via e-mail, de-duplicated and consolidated into one list. The list of seminal articles was compared with the articles included in the RRNA, and articles excluded due to non-accessibility. The clinical research priorities were compared with those identified by the CT from the RRNA data.

## Results

### Process

The CT reviewed and updated the protocol as appropriate for the LF outbreak scenario and submitted it to the information specialist and all systematic review teams on day 1. The information specialist completed the search and submitted the results as an Endnote library to the systematic review teams on day 1 (Table 1).

**Table 1:** The RRNA pilot progress from day 1 to 5

Team	Day 1	Day 2	Day 3	Day 4	Day 5
Coordinating team (CT)	❖ Pilot triggered ❖ Protocol reviewed, updated, and submitted to information specialist and SRT		❖ Manual retrieval of full-text articles ❖ Full-text articles uploaded to Dropbox as pdfs	❖ Manual retrieval of full-text articles ❖ Full-text articles uploaded to Dropbox as pdfs	❖ Data collated and incorporated into the final report at the end of day 5
Information specialist	❖ Search completed ❖ Search result submitted to the SRT as Endnote file	❖ Automatic full-text article retrieval (Endnote) ❖ Full-text articles submitted as pdf’s or URLs			
Systematic review teams (SRT)	❖ Title and abstract screening	❖ Title and abstract screening ❖ Full-text screening	❖ Full-text screening ❖ Data extraction	❖ Full-text screening ❖ Data extraction	❖ Full-text screening ❖ Final resolution of conflicts ❖ Data extraction ❖ Data extraction table and associated information submitted to the CT via e-mail

Screening of title and abstracts began before the end of day 1, started by the review team in Canada, followed by the Philippines, then the UK, optimizing use of global time zone, and was completed by day two, with 39% (*n* = 428/1104) of records passing this step and requiring further assessment. In parallel to the screening of title and abstracts, the information specialist retrieved full-text papers using Endnote’s automatic retrieval function. This was complemented by a manual search of papers not retrieved automatically. The full-text papers were submitted to the review teams and uploaded to DistillerSR in sections. Using the software allowed review processes to be carried out in parallel, while reducing the need for handovers. As soon as papers were deemed potentially eligible, another reviewer could start the full-text screening followed by data extraction, while others continued screening of title and abstracts (Table 1). In tandem, electronic copies of journal articles could be uploaded in the system, and progress tracked in real time. Issues were dealt with via the online instant messenger group and during a brief daily mid-day Skype meeting between the review teams. Two members of the CT were on standby to assist throughout the 5 days.

The full-text screening started on day 2, but the final conflicts were not resolved until early on day 5 (acceptance rate 28% (*n* = 118/428). Of the included articles, the study design was identified for 93% (*n* = 110/118) within the time frame. After study design prioritization, 47% (*n* = 52/110) of the included articles with study design identified, qualified for data extraction. Most of these (88% (*n* = 46/52)) were extracted by mid-day on day 5.

The search was completed earlier than planned, which meant that the screening of title and abstracts and full-text papers commenced earlier than scheduled. However, the retrieval and screening of full-text papers and resolution of conflicts took longer than anticipated. This was partly due to the higher than expected number of articles identified for a (re-) emerging infectious disease and because Endnote’s automatic paper retrieval only retrieved 51% (*n* = 565/1104) of the articles as PDFs (*n* = 436) or URL links (*n* = 129). The remaining full-text papers, which passed through the first title and abstract screening step (*n* = 249), were searched for manually by members of the CT through the University of Oxford online library access. Papers not found after the manual search (*n* = 145) were excluded due to inaccessibility, instead listed with full bibliography in the final report (Additional file 2). Most of these articles were not accessible due to being published in non-English languages and journals. There were no systematic reviews or randomized controlled trials (RCTs) identified amongst the non-accessible articles. The data extraction table was submitted to the CT as a Microsoft Excel file by mid-day on day 5. This was supplemented by a PRISMA flow chart and bibliographies of the studies which were excluded due to not being accessible within the time frame (Additional file 2) or included but not being data extracted (Additional file 3). The unexpected retrieval and resolution of full-text article delays meant that the proportional check of the extracted data by a second reviewer was not completed within the 5 days. Instead, a member of the CT checked 30% of the extracted data post-day 5.

### Outcomes and gaps in evidence

The data extracted were comprehensive, as would be required for a systematic review. However, for the purpose of the RRNA, in order to enable identification of gaps in evidence and knowledge in a short timeframe, the data were streamlined by a member of the CT to highlight the key PICO (Population, Intervention, Comparison, Outcome) parameters required for this purpose, in addition to bibliography, study design, and setting. Data queries were resolved via e-mail after completion of the pilot. Due to the high number of articles identified and the amount and complexity of data extracted, tidying and organizing of the data to facilitate identification of gaps in evidence took longer than expected. The final report was submitted to the steering group, together with a PRISMA flowchart and the supplements 3 days post-pilot completion. Gaps in evidence were identified, individually by members of the CT, through a review of the extracted data for each clinical domain. A narrative summary of the level of evidence and gaps in evidence identified from the data by clinical domains is provided below.

### Characteristics of the included studies

**Fig. 3:**
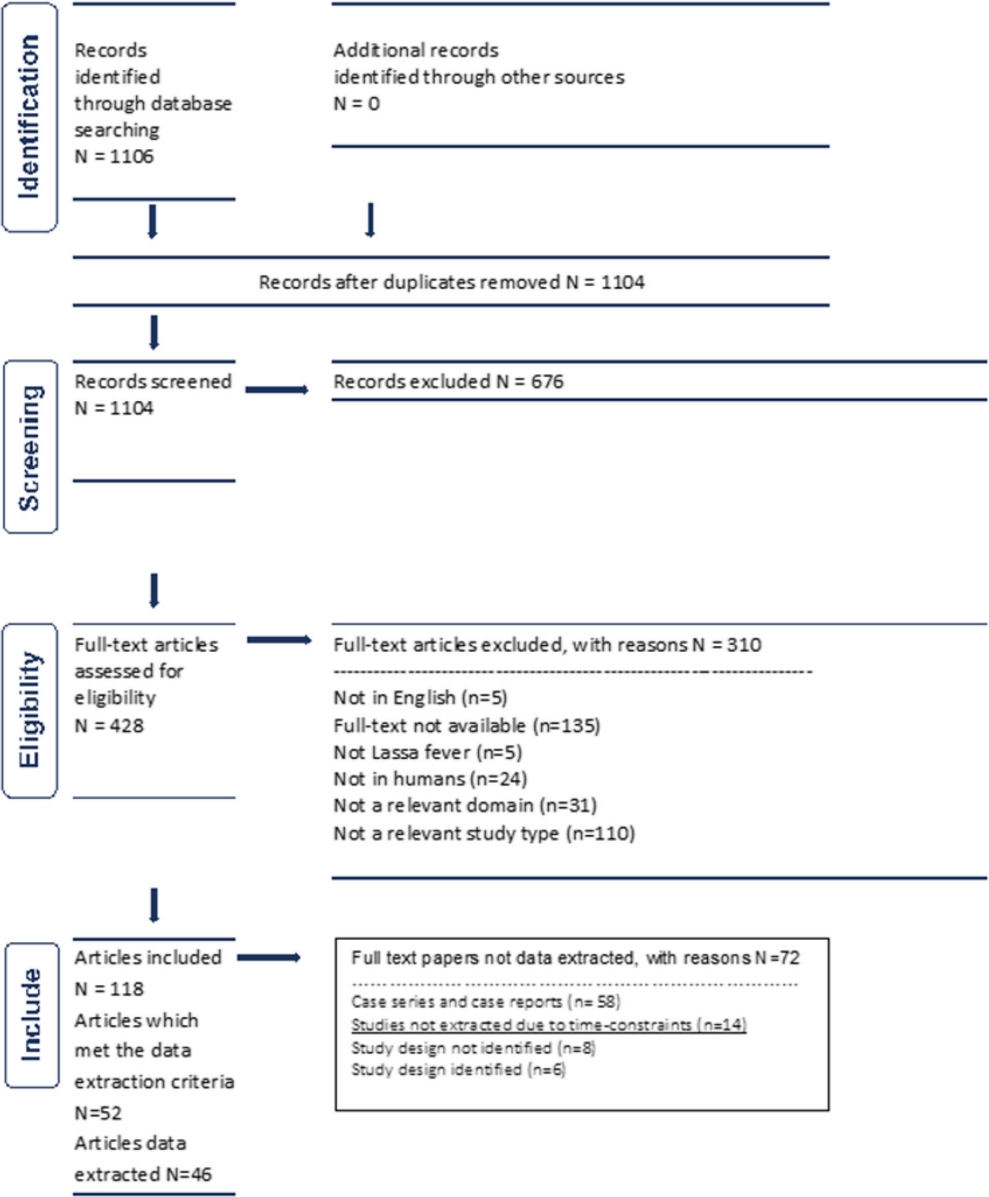
PRISMA flowchart

The electronic literature search identified 1104 records, published between 1969 and July 2017, of which 118 met the inclusion criteria (Fig. 3). Study design and clinical domains addressed in the articles were identified for 110 of the articles. There were no systematic reviews, meta-analysis, or RCTs identified. Two were non-randomized controlled studies, 34 cohort studies (some of which included before and after comparisons), 4 case-control studies, 12 cross-sectional studies, 27 case series, and 31 case reports (Table 2).

**Table 2:** Type of study designs identified for each clinical domain

Domain	Study design					
	Non-randomized controlled studies	Cohort studies	Case-control studies	Cross-sectional studies	Case series^#^	Case report^#^
Clinical phenotype and natural history of disease	1 article [18]	18 articles* [19–38]	3 articles [39–41]	7 articles [42–48]	21 articles [49–69]	27 articles [70–96]
Transmission and prevention		10 articles** [22, 29, 38, 97–103]		3 articles [42, 43, 104]	3 articles [61, 62, 105]	7 articles [73, 74, 83, 84, 91, 92, 106]
Diagnostics		5 articles** [19, 37, 107–109]	1 article [110]	2 articles [46, 111, 112]	1 article [69]	4 articles [55, 74, 82, 113]
Immune response		4 articles** [31, 32, 107, 114]		2 articles [44, 46]	6 articles [52, 65, 115–118]	9 articles [55, 70, 72, 73, 76, 86, 91–93]
Drug therapy and supportive care	1 article [119]	7 articles** [19, 21, 24, 38, 120–122]		3 articles [42, 123, 124]	8 articles [49, 51–53, 56, 64, 66, 125]	17 articles [55, 71, 73–78, 80, 81, 83, 85, 86, 89, 95, 96, 126]
Risk factors for more severe disease		3 articles [37, 38, 114]		1 article [44]		

*4 not extracted

**1 not data extracted

^**#**^Not extracted since higher level of evidence available for the domains covered in each article

The articles were prioritized so that data were extracted first from studies whose design provided the highest level of evidence. Ongoing studies and conference abstracts were also prioritized, since they can present important preliminary findings. Case series and case reports were not extracted when higher level of evidence was available for all the clinical domains addressed in the article. Of the 110 articles, 52 met the data extraction criteria and 46 were extracted within the 5 days [18–27, 32–34, 36–48, 97–104, 107, 109–112, 114, 119, 121–124, 127]. Twenty-seven case series and 31 case reports were not extracted due to higher level of evidence being available for the domains addressed in the articles. Six cohort studies [28–31, 108, 120] and eight articles [128–135] without study design identified were not extracted due to resource limitations (Additional file 3). Of the 46 data extracted articles (Additional file 4), most were set-in low-income countries in West Africa: Sierra Leone (*n* = 19), Nigeria (*n* = 17), Liberia (*n* = 5), Guinea (*n* = 2), and Mali (*n* = 2). Five were indicated as set-in high-income countries: the USA (*n* = 3), Germany (*n* = 2), and the UK (*n* = 1). Three articles presented studies set in more than one country [48, 114, 121]. Five were conference abstracts [33–36, 41] and two ongoing cohort studies registered by the US Army, evaluating effectiveness and safety of ribavirin, expected to be completed in 2018 and 2019 [121, 122]. Table 3 presents an overview of the study setting, populations, objectives, and interventions identified for the studies in each clinical domain.

**Table 3:** Overview of the included studies

Domain	No. of extracted studies	Setting*	No. of participants	Populations	Study objectives	Intervention
Clinical phenotype and natural history of disease	25	14 Nigeria 7 Sierra Leone 3 Liberia 3 Mali 1 USA	Total: *n* = 6680 LF pos. *n* = 1734	Adults Pregnant women Children, Infants, Neonates (0 to > 65 years old)	Clinical presentation, symptoms (*n* = 15) LF fatality rate (*n* = 11) Biochemical laboratory parameters (*n* = 7)	N/A
Transmission and prevention	12	5 Sierra Leone 4 Nigeria 1 Germany 1 Liberia 1 UK 1 USA	Total: *n* = 1980 LF pos: *n* = 281	Adults Pregnant women Children, Infants, Neonates (0 to 73 years)	Ribavirin as PEP (*n* = 4) Risk of nosocomial transmission (*n* = 5)	Ribavirin
Diagnostics	7	4 Sierra Leone 2 Liberia 1 Nigeria	Total: *n* = 3338 LF pos: *n* = 897	Adults Children	PCR for diagnostics (*n* = 3) PCR and hybridization (*n* = 1) LFI, ELISA and PCR (*n* = 1) IgM as early marker (*n* = 1)	PCR, LFI, ELISA, virus isolation
Immune response	5	2 Guinea 2 Sierra Leone 1 Liberia 1 Mali	Total: *n* = 4570 LF pos: *n* = 1437	Adults Children, Infants (7 months to 83 years)	Levels of inflammatory cytokines chemokines and other pro-inflammatory mediators (*n* = 1) Prevalence of LASV-specific IgG antibodies (LV IgG) (*n* = 1) Population LF seroconversion (*n* = 1)	
Drug therapy and supportive care	10	4 Nigeria 4 Sierra Leone 2 USA 1 Germany	Total: *n* = 1516 LF pos: *n* = 792	Adults Pregnant women Children, Infants, Neonates (0 to 65 years)	Therapeutic effectiveness of Ribavirin (*n* = 9) Therapeutic effectiveness of LF convalescent plasma therapy (*n* = 2) Ribavirin treatment adverse event (*n* = 1)	Ribavirin iv. Ribavirin oral Convalescent plasma
Risk factors for more severe disease	4	3 Sierra Leone 2 Guinea	Total: *n* = 2110 LF pos. or probable: *n* = 562	Adults Pregnant women Children, Infants, Neonates (0 to > 60 years)	Correlation of cytokine levels and outcome (*n* = 2) Correlation of AST and outcomes (*n* = 1) Correlation of BUN, ALP, ALT, and outcomes (*n* = 1) Correlation of viremia level and outcome (*n* = 1) Risk factors for positive LASV IgG (*n* = 1)	

*Abbreviations*: *LF* Lassa fever, *LASV* Lassa virus, *Pos* positive, *PEP* post-exposure prophylaxis, *PCR* polymerase chain reaction, *LFI* lateral flow immunoassay, *ELISA* enzyme-linked immunosorbent assay, *Ig* immunoglobulin, *AST* aspartate aminotransferase, *BUN* blood urea nitrogen, *ALP* alkaline phosphatase, *ALT* alanine aminotransferase

*Some studies were set in more than one country

#### Clinical phenotype and natural history of disease

Seventy-seven articles identified addressed this domain (Table 2). After prioritization by study design, 29 articles published from 1975 to 2017 qualified for data extraction, of which 25 were extracted within the time frame. These articles reported data on almost 7000 people, representing more than 1700 confirmed LF cases (Table 3). Most of the studies were set in West Africa. Fourteen studies were set in Nigeria [18, 19, 21, 22, 24, 26, 33, 34, 36, 39, 41, 42, 48, 127], seven in Sierra Leone [20, 25, 27, 40, 43, 45, 48], three in Liberia [23, 46, 48], three in Mali [32, 47, 48], and one in the USA [48]. One study was set in multiple locations [48]. The five conference abstracts identified related to studies in this domain. Four cohort studies published from 1974 to 2001 [28–31] were not extracted due to resource limitations (Additional file 3).

Symptoms of LF on presentation and during hospital admission in healthcare settings in West Africa were described in 15 studies [19, 21, 23–27, 33, 35, 36, 39–41, 45, 48]. The data shows that LF has been studied in adult and pediatric populations in lower income healthcare settings in West Africa. Most of the studies were observational studies, and many lacked data on case definition, diagnostic criteria used to support the findings, or risk factors, such as comorbidities or immunosuppression through age, illness, or medication. Another gap identified was the quantification of the risk of complications, more severe disease, and sequelae in different at-risk populations. Eleven studies presented data on CFR with standard care: six were set in Nigeria [22, 26, 33–35, 39], three in Sierra Leone [25, 27, 43], and two in Liberia [23, 46]. The CFR ranged from 5.6 to 75%, but with limited information to explain the wide variations observed in different settings or description of the standard care provided.

#### Transmission and prevention

Twenty-three articles were identified for this domain; 13 qualified for data extraction and data were extracted for 12 of these, reporting data from 1980 participants, including nearly 300 confirmed cases of LF (Tables 2 and 3). The studies were set in Sierra Leone (*n* = 5) [38, 43, 98, 100, 103], Nigeria (*n* = 4) [22, 42, 101, 102], Liberia (*n* = 1) [104], Germany (*n* = 1) [99], the UK (*n* = 1) [97], and the USA [103]. One study was set in more than one country [103]. Several of the studies reported risk of nosocomial transmission to other patients and healthcare workers [22, 38, 42, 43, 104], with attack rates ranging from 11 to 55% in different settings [22, 104]. Four studies reported the use of ribavirin as post-exposure prophylaxis (PEP) [98, 100–102], administered to a different level of contacts. A total of 64 Lassa fever contacts were treated with the drug. Though it was evident that there is a risk of transmission from body fluids in hospital settings, there were no robust studies identified studying risk of transmission from different types of body fluids or organs, moreover, a lack of standardization of definitions of a “contact.”

#### Diagnostic

Fourteen studies were available for this domain; eight qualified for data extraction, and seven were extracted within the time frame (Table 2). Four of the extracted studies were set in Sierra Leone [37, 107, 109, 111], two in Liberia [46, 112], and one in Nigeria [110], providing data from more than 800 confirmed cases (Table 3). Most identified studies were observational, with one case-control study. The studies used different diagnostic tests, with polymerase chain reaction (PCR) the most commonly used. Neither a gold standard diagnostic test nor clinical or laboratory case definitions were reported. Moreover, systematic diagnostic and sampling strategies were not reported. The studies indicated that no single diagnostic test could detect all cases or strains of LF [109, 111, 112].

#### Immune response

Twenty-one studies were identified for this domain; six articles qualified for data extraction of those five were extracted within the time frame (Table 2). Two studies were set in Sierra Leone [107, 114], two in Guinea [44, 114], one in Mali [32], and one in Liberia [46]. One study was set in more than one country [114]. Though there were a limited number of studies identified, they provided data from more than 4500 participants, including 1400 LF-positive people (Table 3). One study reported seroprevalence data from a rural region of Guinea [44], and a cohort study reported that LF IgM antibodies may persist for months to years [107]. The data shows that there are large gaps in evidence regarding the immune response to LF infection caused by different strains, in different at-risk populations and over time.

#### Drug therapy and supportive care

Of 36 articles identified for this domain, 11 qualified for data extraction and ten were extracted within the time frame (Table 2). Four studies were set in Sierra Leone [38, 119, 123, 124], four in Nigeria [19, 21, 24, 42], and two ongoing studies by the US Army [121, 122] (set in the USA and Germany), providing data for more than 1500 participants and almost 800 LF cases (Table 3). The highest level of evidence in this domain was a non-randomized controlled study set in Sierra Leone (1986, *n* = 312), which studied the effect of ribavirin and convalescent plasma therapy. It was a relatively small study, which showed no reduction in case fatality rates using LF convalescent plasma, but indicated that ribavirin was effective, especially if administered within the first 6 days of illness [119].

#### Risk factors for more severe disease

There were four studies identified and data extracted for this domain (Table 2). The studies were set in Sierra Leone [37, 38, 107, 114] and Guinea [44, 114]. One study was set in both countries [114]. The data represented more than 2000 participants and over 500 confirmed or probable LF cases (Table 3). A number of studies reported that elevated aspartate aminotransferase levels were associated with increased mortality rates [37, 38, 107], one study also reported a correlation between low levels of blood urea nitrogen, alkaline phosphatase, and alanine aminotransferase and survival [107]. Two studies reported an association between low levels of a number of cytokines and survival rates [107, 114].

### Pilot evaluation

#### Stakeholder pilot evaluation

Some of the key facilitators and barriers identified by the stakeholders taking part in the pilot, through a short post-LF pilot survey and subsequent telephone conference, are presented in Table 4. The evaluation highlighted that previous training in the methodology and experience of systematic review methods were key facilitators. Moreover, that use of the online systematic review software and global time zones was an effective way of optimizing resources. The barriers identified also highlights the need to ensure that everyone is familiar with all the systems used, and in identifying sufficient resources at the outset of the outbreak, tailored to the type of outbreak and number of articles identified after the initial search.

**Table 4:** Protocol facilitators and barriers identified

Facilitators	Barriers
• Review teams with previous experience of systematic and rapid reviews involving clinical research was a key facilitator for protocol development and piloting • An experienced information specialist for developing and carrying out a rapid, robust search strategy • Engaging stakeholders involved in the pilot in the development of the protocol ensured all were trained in the methodology in advance • The brief clinical LF background data summarized by the CT were submitted to all on day 1 • The “global relay” set up in advance, which optimized the use of time zones and resources • The use of DistillerSR allowed the organization of the data and different steps to be carried out in parallel. It also reduced the need for handovers, though the reviewers found that a brief, daily handover meeting was useful • The use of an instant messenger system aided the rapid response to specific queries • The CT on stand-by as extra resources was helpful in order to respond to clinical queries and assisting with full-text paper retrieval and consensus	• Endnotes’ automatic retrieval of full-text articles was not as effective as expected. This meant that additional resources had to be identified rapidly to assist with retrieving full-text papers, causing unforeseen delays • The higher than expected number of articles identified meant that resources were stretched to capacity • Screening of full-text papers took longer than expected • The reviewers found some of the clinical domains, such as diagnostics and immune response harder to review and data extract • The large number of articles identified also meant that there were not enough resources to translate non-English papers • One review team not having access to Endnote during the pilot • The large amount of data extracted meant that it took longer than anticipated to tidy and organize the data

#### Clinical research priorities

A comparison of the clinical research priorities identified by the steering group from the RRNA data with the priorities independently identified by the LF expert panel shows that though there is overlap, each method identified unique priorities. Despite the number of articles identified by the RRNA, the types of studies that were reported meant that there was limited robust evidence in the clinical domains investigated with inadequacies in the quality of the reporting (such as a lack of case definitions and methods used for diagnostics). This meant that the steering group, through a review of the extracted data, identified several clinical research priorities where important uncertainties and evidence gaps remain. These are presented in the table, together with the clinical research priorities identified by the independent LF expert panel (Table 5). Of the 19 clinical research priorities informed by the RRNA data, 11 of those were also listed as priorities by the expert panel, together with an additional ten research priorities. This shows that the RRNA methodology was able to identify most of the priorities identified by the Lassa fever expert panel, together with an additional eight unique priorities not identified by the expert panel. The priorities identified by the expert panel tended to be more specific, whereas the priorities identified from the RRNA covered a wider focus.

**Table 5:** Lassa fever clinical research priorities identified

Clinical phenotype and natural history of disease	RRNA	Expert panel
Which are the populations at risk?	✓	✓
What is the true incidence of asymptomatic infection; is the reported 85% of asymptomatic infections true or is there a diversity of clinical presentation?		✓
What are the clinical characteristics of Lassa fever in different at-risk populations?	✓	
What are the long-term health sequelae and what is their frequency, severity, and duration?	✓	✓
What are the underlying pathophysiological mechanisms of death and are these preventable e.g. acute kidney injuries? What is the cause of platelet dysfunction in acutely ill Lassa patients?	✓	✓
What is the clinical and epidemiology relevance of Lassa virus sequence heterogeneity?		✓
Transmission and prevention		
What are the risks of person-to-person transmission associated with different types of exposure e.g. to what extent and how does human-human transmissions account for disease transmission? What is the risk of transmission from different body fluids and organs?	✓	✓
Does disease severity vary with route of transmission?	✓	
Does genetic differences within and between Lassa virus strains results in differences in transmission and in disease phenotype?		✓
Who are the target population for a Lassa vaccine, e.g. does asymptomatic infection protect against re-infection? Does presence of antibodies protect from re-infection?	✓	✓
Does ribavirin PEP reduce the risk of Lassa virus disease, or more severe disease?	✓	
What is the optimal route and dosing for post-exposure prophylaxis with ribavirin (e.g. oral vs. intravenous)?	✓	✓
How diverse does a vaccine need to be to protect against all strains of Lassa virus?		✓
Diagnostics		
Can we develop a diagnostic test that is highly sensitive and specific for all lineages?	✓	✓
How does sequence variation/heterogeneity impact diagnostic methods and accuracy?		✓
What is the optimal sampling time frame for diagnostics using RT-PCR? How many days after symptoms does Lassa virus become detectable by PCR?	✓	
Can we develop a validated point-of-care test for use in different healthcare settings, including rural health posts?	✓	✓
Immune response		
What are the dynamics of resistance to re-infection? What is the average kinetics of antibody responses following acute Lassa fever virus infection and what is the variability between individuals and by age?	✓	✓
In what sites and for how long does virus persist? What are the risk factors for virus persistence?		✓
Does previous exposure to Lassa virus result in more severe disease upon subsequent re-exposure (e.g. vaccine) as a result of antibody-dependent enhancement of infection, i.e. could a vaccine do harm?		✓
What immunological end-points should be used for Lassa virus vaccine trials?	✓	
Drug therapy and supportive care		
What is the true efficacy and safety of ribavirin for the treatment of Lassa? Can we transition acutely ill Lassa patients to oral ribavirin once viral loads are decreasing?	✓	✓
Does the use of ribavirin in acute Lassa fever virus infection improve clinical outcomes compared to supportive care alone?	✓	✓
What is the optimal approach to supportive care for acutely ill patients with Lassa and other VHFs?		✓
What is the target therapeutic plasma and CSF concentrations of ribavirin for the treatment of Lassa fever virus infection? Do current oral and IV treatment regimens achieve these target concentrations?	✓	
Can type 1 interferon therapy boost the efficacy of ribavirin? Is there a role for therapies directed at host immunopathology in the management of Lassa fever?		✓
Risk factors for more severe disease		
Are reported differences in CFR attributable to differences in case mix (e.g. illness severity on presentation to a healthcare facility), differences in the underlying prevalence of risk factors for death, or differences in the care provided?	✓	
Are there clinical features or biomarkers of the risk of progression to severe disease that have clinical utility?	✓	
Do genetic differences within and between Lassa strains results in differences in disease phenotype and disease severity?		✓

#### Seminal articles

There were 21 seminal articles identified by the LF expert panel. These were all identified by the RRNA search strategy, although 13 did not meet the inclusion criteria for the RRNA and were excluded at the screening stage, mainly due to being animal or cell culture studies. The remaining eight articles [25, 44, 60, 62, 63, 65, 96, 119] were all included in the RRNA. Three of these articles [25, 44, 119] met the data extraction criteria and were extracted. The other five [60, 62, 63, 65, 96] were case series or case reports and not extracted, instead included with full bibliography. This shows that the RRNA successfully identified and included all seminal articles identified by the LF expert panel which met the RRNA inclusion eligibility criteria.

## Discussion

The development and piloting of the RRNA process demonstrates that a global partnership, through effective use of time zones, can deliver a robust summary of much of the published clinical research evidence on Lassa fever within 5 days in response to an outbreak. The results show that the RRNA methodology can be a valuable tool for rapidly identifying gaps in evidence and informing clinical research priorities in response to (re-) emerging outbreaks globally, ideally combined with an expert panel for further refinement of the research priorities.

Key strengths of the process were the use of experienced systematic reviewers, trained in the RRNA methodology in advance and based across different global time zones for resource optimization. Use of an online systematic review software allowed various steps in the review process to be carried out in parallel, minimizing handovers and knowledge transfer loss. The RRNA protocol was developed specifically for emerging outbreaks where previous clinical research is likely limited. Therefore, the protocol was designed to be over-inclusive at the screening stage, with the aim to reduce the risk of any relevant studies being missed. However, for the LF pilot, the number of articles retrieved and deemed eligible was higher than expected from previous experiences for a re-emerging outbreak, which meant that the full-text screening and data extraction took longer than anticipated. This was alleviated by the prioritization of the data extraction step, whereby lower levels of evidence were not extracted if a higher level of evidence were identified for the clinical domains covered. Furthermore, articles in languages other than English were included but not data extracted. For the future, we recommend that resources should be allocated in relation to the number of articles identified at the search stage. Moreover, depending on the nature of the outbreak and region of endemicity of the pathogen, reviewers with appropriate language skills can be identified at the outset.

In this pilot, the reviewers found that the diagnostics and immune response domains were more difficult to process, in regard to assessing which articles to include and relevant data to extract. This delayed the reaching of consensus at the full-text screening stage and the data extraction. Engaging reviewers with relevant content expertise as well as those with expertise in systematic review screening and data extraction processes, and ensuring understanding of all domains in advance, could help with the speedier resolution of queries and identification of the key data to extract.

Despite the high number of articles identified, the RRNA was able to identify key clinical research articles, which enabled the CT to identify a broad range of gaps in evidence and clinical research priorities across all domains, including eight unique research questions not identified by the expert panel. Prospective clinical observational studies of patients with Lassa fever could answer many of the key evidence gaps, such as risk factors and pathophysiological mechanisms for severe disease, infectivity of body tissues, long-term sequelae, and immunological responses. A need for diagnostic studies was also identified by both the RRNA method and the experts and could be nested within clinical observational studies. In addition, clinical trials are needed to evaluate the use of supportive care strategies, ribavirin, and other potential therapeutics. Given the fairly predictable timing and location of LF cases, all these research questions should be tractable given sufficient resources and effort.

Although there was an overlap of research questions identified by the RRNA team and the panel members, both methods identified unique research questions. The questions identified by the RRNA team tended to be broader, whereas the expert panel identified more focused questions. This shows that using both methods can lead to a more robust and comprehensive set of research priorities that covers all domains, while ensuring specificity, than relying on either process alone. All seminal papers that fit the inclusion criteria were identified by the RRNA methodology. The number of seminal papers highlighted by the panel that did not fit the inclusion criteria emphasizes the need to ensure that all stakeholders are fully trained in the process in advance, ideally through a briefing meeting to ensure full understanding of the process.

The pilot evaluation shows that it is feasible to carry out a robust RRNA within 5 days in response to an outbreak, to inform clinical research priorities. Another 3 to 5 days are recommended for summarizing and reviewing the extracted data, in order to identify gaps in evidence and to formulate clinical research priorities. The resource limitations encountered can be minimized by identifying reviewers, depending on the number of articles identified at the search stage. Depending on the nature of the outbreak, e.g., endemicity, reviewers with specific language skills should be engaged. Identifying experts for the panel took longer than anticipated and although the clinical researchers who formed the expert panel for this pilot had years of experience in LF research, in collaboration with research groups in endemic areas, engaging experts from endemic areas would be desirable. For sustainability, it is important to identify a pool of clinical researchers and systematic reviewers globally, with a focus on infectious disease “hot spot” regions, that are trained in the process and on “stand-by” ready to be activated. Moreover, it is important to engage a pool of global emerging infectious disease experts in advance. The fact that the time commitment is limited minimizes the time spent away from other work commitments and can act as an important facilitator. Timely and effective data sharing during a public health emergency and potential copyright issues around sharing of non-open access articles are other challenges that need to be addressed in advance.

## Conclusions

This pilot study shows that the RRNA methodology can be used to systematically and transparently identify important research gaps, in days and weeks rather than months or years. The findings highlight that using a RRNA together with input from a disease-specific expert panel can ensure that a wider range of clinical research priorities is identified than if using either method on its own. The pilot illustrates the benefit of global research networks, but also a need to strengthen networks in epidemic “hot spot” regions, which could ensure people with appropriate language skills and contextual expertise are trained and engaged in future RRNAs. The RRNA is not intended to replace systematic reviews, or to generate data for a meta-analysis, but as a tool to inform rapid research prioritization when there are no systematic reviews or robust RCTs available, such as for many of the WHO priority pathogens [14]. As well as expanding to other diseases areas, it is also important that the gaps that a RRNA identifies for a particular disease are re-visited periodically and in the context of the outbreak. For instance, for Lassa fever, this might include the need for additional research on circulating strains, rate of infectivity, risk groups, risk of transmission from body fluids, re-infection, and health outcomes. These results show that the RRNA can fill in gaps not identified by the expert panel, and the expert panel can help refine the research priorities further. The RRNA methodology can be used in response to any (re-) emerging outbreak globally, when there is no recent systematic review available and applied for the scenario of a “disease X” [14] epidemic.

Box 1 Search terms Embase, PubMed:

We used search terms for “Lassa fever” in text words and controlled vocabulary, in conjunction with terms to define the different questions (domains): incubation, symptom*, “natural history”, “clinical features”, transmission, infectiousness, vaccin*, prophyla*, chemoprophyla*, prevention, protection, diagnosis, diagnostic, RDT*, screening, detection, immunity, serology, treatment, management, therapy, drug*, intervention*,“supportive care”, fluid*, electrolyte*, supplement*, mortality, death, “adverse events”, “side effect*”, complications, sequela*. For all other sources we only used search terms for Lassa fever or Lassa virus.

Cochrane Library, DARE, Epistomonikos, Prospero, Clinical trial registries (clinicaltrials.gov, ISRCTN registry), WHO), CDC and ECDC:

“Lassa fever” or “Lassa virus”

## Additional files

Additional file 1: Clinical research domains. (PDF 94 kb)

Additional file 2: Articles not included due to non-accessibility. (PDF 218 kb)

Additional file 3: Articles for which data were not extracted. (PDF 163 kb)

Additional file 4: Data extraction summary overview. (PDF 191 kb)

## Abbreviations

ALP: Alkaline phosphatase
ALT: Alanine aminotransferase
AST: Aspartate aminotransferase
BUN: Blood urea nitrogen
CDC: The Centers for Disease Control and Prevention
CFR: Case fatality rate
CT: Coordinating team
ECDC: The European Centre for Disease Prevention and Control
ELISA: Enzyme-linked immunosorbent assay
ICTRP: The WHO International Clinical Trials Registry Platform
Ig: Immunoglobulin
ISP: Information specialist
LASV: Lassa virus
LF: Lassa fever
LFI: Lateral flow immunoassay
PCR: Polymerase chain reaction
PEP: Post-exposure prophylaxis
Pos: Positive
RCT: Randomized controlled trials
RRNA: Rapid research needs appraisal
SG: Steering group
SRT: Systematic review team
UK: The United Kingdom
WHO: The World Health Organization
