# Supplementary material for: A rapid research needs appraisal methodology to identify evidence gaps to inform clinical research priorities in response to outbreaks—results from the Lassa fever pilot
Source: BMC Med. 2019 Jun 11;17:107. doi: 10.1186/s12916-019-1338-1 (PMC6560772; doi:10.1186/s12916-019-1338-1)
Supplement: Supplementary file 1 — Clinical research domains. (PDF 94 kb) [file 12916_2019_1338_MOESM1_ESM.pdf]

Additional file 1. Clinical research domains

Domain	Question(s)	Population
Clinical phenotype and natural history of disease	What are the signs and symptoms of the disease?<br>What are the laboratory (haematology, biochemistry, coagulation etc.) features of disease?<br>Which constellations of clinical features distinguish disease from differential diagnoses?<br>Are there distinct clinical syndromes amenable to staging/grading?<br>Does asymptomatic infection occur?<br>What is the mortality rate?	Neonates<br>Infants<br>Children<br>Adults<br>Elderly<br>Pregnant<br>Breastfeeding<br>Immunosuppressed<br>Malnourished<br>Comorbidities
Transmission and prevention	What is the incubation period of the disease?<br>What are the routes of transmission?<br>What are the infective body fluids? When and how long are they infectious for?<br>How effective is vaccination (if it exists) at preventing disease?<br>What are the side effects of vaccination?<br>How effective is drug prophylaxis (if it exists) at preventing disease?<br>How effective is post-exposure drug prophylaxis (if it exists) at preventing disease?<br>What are the side effects of drug prophylaxis?	
Diagnostics	What is the sensitivity and specificity of different diagnostic tests? In different bodily fluids (e.g., blood, CSF, urine)?	
Immune response	What is the serological response to infection?	
Drug therapy and supportive care (e.g., electrolytes, fluids)	What is the effect of supportive care on:<br>1. Length of hospital stay?<br>2. Complications<br>3. Mortality rate?<br>What is the effect of different doses, routes and frequencies of supportive therapy on the response?<br>What are the side effects of supportive therapy	
Risk factors for more severe disease	What are the risk factors for disease?<br>What are the risk factors for severe disease?<br>What are the risk factors for mortality?<br>What are the risk factors for long term complications/sequela?	
