# Supplementary material for: A rapid research needs appraisal methodology to identify evidence gaps to inform clinical research priorities in response to outbreaks—results from the Lassa fever pilot
Source: BMC Med. 2019 Jun 11;17:107. doi: 10.1186/s12916-019-1338-1 (PMC6560772; doi:10.1186/s12916-019-1338-1)
Supplement: Supplementary file 2 — Articles not included due to non-accessibility. (PDF 218 kb) [file 12916_2019_1338_MOESM2_ESM.pdf]

Additional file 2. Articles not included due to non-accessibility

Full-text article not available (n=145)

This list includes papers that were searched for manually in the University of Oxford library.

Bibliography
Anonymous. [Lassa fever]. *Orv Hetil.* 1976. 117:1580-4 [Hungarian]
Anonymous. Communicable disease report United Kingdom--January to March 1982. *Community Med.* 1982. 4:238-41
Anonymous. Lassa fever. *Med Leg J.* 1976. 44:107-8
Adomajlo, A.. [Epidemiology of Lassa fever]. *Przegl Epidemiol.* 1976. 30:243-6
Akoua-Koffi, C.,Ter Meulen, J.,Legros, D.,Akran, V.,Aidara, M.,Nahounou, N.,Dogbo, P.,Ehouman, A.. [Detection of anti-Lassa antibodies in the Western Forest area of the Ivory Coast]. *Med Trop (Mars).* 2006. 66:465-8
Anusz, A.. [Lassa fever]. *Pieleg Polozna.* 1981:7
Ayoola, E. A.. Infectious diseases in Africa. *Infection.* 1987. 15:153-9
Bangura, J. J.,Fair, J.,Goba, A.,Khan, S. H.,Fonnie, R.,Garry, R. F.,Moses, L. M.,Nylander, F.,Daboi, J.,Kpoto, L.,Lugala, P. C.,Koivogui, L.,Coulibaly, M.,Lamunu, M.,Roth, C.,Bausch, D. G.. Epidemiology of lassa fever in the mano river union countries of West Africa, 2004-2008. *American Journal of Tropical Medicine and Hygiene.* 2009. 1):209-210
Bausch, D. G.,Rollin, P. E.. Lassa fever. [French]
Bengtsson, E.. [Lassa fever--a new and contagious exotic imported disease]. *Lakartidningen.* 1976. 73:3425-6
Bergmann, J. F.. New African viral fevers Ebola, Lassa and Marburg. [French]
Best, E. W.. The Lassa fever episode, Metro Toronto, August, 1976. *Can J Public Health.* 1976. 67:361-6, 369-74
Boiro, I.,Lomonossov, N. N.,Sotsinski, V. A.,Constantinov, O. K.,Tkachenko, E. A.,Inapogui, A. P.,Balde, C.. [Clinico-epidemiologic and laboratory research on hemorrhagic fevers in Guinea]. *Bull Soc Pathol Exot Filiales.* 1987. 80:607-12
Borisevich, I. V.,Markin, V. A.,Firsova, I. V.,Evseev, A. A.,Khamitov, R. A.,Maksimov, V. A.. [Hemorrhagic (Marburg, Ebola, Lassa, and Bolivian) fevers: epidemiology, clinical pictures, and treatment]. *Vopr Virusol.* 2006. 51:8-16
Borkowski, W.. [Lassa fever (author's transl)]. *Przegl Lek.* 1974. 31:868-70
Bouree, P.. [Lassa fever and ribavirin]. *Med Sante Trop.* 2015. 25:130
Bowell, E.. Journal of Infection Control Nursing. Nursing the isolated patient: Lassa fever. *Nurs Times.* 1986. 82:72-81
Bres, P.. [Recent Lassa, Marbourg and Ebola viruses in African tropical viruses. I. Semiology--physiopathology--diagnosis--treatment (author's transl)]. *Nouv Presse Med.* 1978. 7:2921-6
Brudnjak, Z.,Babic, K.. [Lassa fever (author's transl)]. *Lijec Vjesn.* 1977. 99:551-6
Buzina, D. S.. LASSA - Brief insight [1]. *Acta Dermatovenerologica Croatica.* 2000. 8:129-130
Casals, J.,Buckley, S. M.. Lassa fever. *Prog Med Virol.* 1974. 18:111-26
CDC. Viral hemorrhagic fever: initial management of suspected and confirmed cases. *MMWR Morb Mortal Wkly Rep.* 1983. 32 S:2:27S-38S
Chen, J. P.,Cosgriff, T. M.. Hemorrhagic fever virus-induced changes in hemostasis and vascular biology. *Blood Coagul Fibrinolysis.* 2000. 11:461-83
Courtois, D.. [Lassa fever]. *Soins Pathol Trop.* 1986:13-5
Cowper-Smith, F.. Research for survival. *Nurs Times.* 1976. 72:1876-8
Coyle, A. L.. Lassa fever. *Nursing.* 2016. 46:69-70
Crotty, S.,Cameron, C.,Andino, R.. Ribavirin's antiviral mechanism of action: lethal mutagenesis?. *J Mol Med (Berl).* 2002. 80:86-95
Crowcroft, N. S.. Management of Lassa fever in European countries. *Euro Surveill.* 2002. 7:50-2
Cummins, D.,Bennett, D.,Fisher-Hoch, S. P.,Farrar, B.,Machin, S. J.,McCormick, J. B.. Lassa fever encephalopathy: clinical and laboratory findings. *J Trop Med Hyg.* 1992. 95:197-201
Cummins, D.,Bennett, D.,Fisher-Hoch, S. P.,Farrar, B.,McCormick, J. B.. Electrocardiographic abnormalities in patients with Lassa fever. *J Trop Med Hyg.* 1989. 92:350-5
Cummins, D.,Fisher-Hoch, S. P.,Walshe, K. J.,Mackie, I. J.,McCormick, J. B.,Bennett, D.,Perez, G.,Farrar, B.,Machin, S. J.. A plasma inhibitor of platelet aggregation in patients with Lassa fever. *Br J Haematol.* 1989. 72:543-8
Cummins, D.. Lassa fever. *Br J Hosp Med.* 1990. 43:186-8, 190, 192
Cummins, D.. Rats, fever and sudden deafness in Sierra Leone. *Trop Doct.* 1992. 22:83-4
Denis, F.,Robin, Y.. [Marburg, Ebole and Lassa virus infections]. *Dakar Med.* 1979. 24:51-5
Eichenlaub, D.,Pohle, H. D.. Haemorrhagic fevers - Risk for hospital staff?. [German]

El-Bahnasawy, M. M.,Megahed, L. A.,Abdalla Saleh, H. A.,Morsy, T. A.. Lassa fever or lassa hemorrhagic fever risk to humans from rodent-borne zoonoses. *J Egypt Soc Parasitol.* 2015. 45:61-70

El-Bahnasawy, M. M.,Megahed, L. A.,Saleh, H. A.,Abdelfattah, M. A.,Morsy, T. A.. TRAINING PROGRAM FOR NURSING STAFF REGARDING VIRAL HEMORRHAGIC FEVERS IN A MILITARY HOSPITAL. *J Egypt Soc Parasitol.* 2015. 45:249-72

Emond, R. T.. Exotic infectious diseases: Lassa fever. *R Soc Health J.* 1980. 100:48-52

Erofeeva, N. I.,Trofimov, N. M.,Surikova, L. E.,Vereshchako, N. S.,Fidarov, F. M.,Petkevich, A. S.. [The comparative characteristics of the genetic traits of the Lassa and Mopeia viruses]. *Vopr Virusol.* 1990. 35:241-3

Fabiyi, A.,Smith, E. A.,Tomori, O.. Lassa fever: clinical symptoms and laboratory diagnosis. *Niger Med J.* 1979. 9:12-6

Fisher-Hoch, S. P.,McCormick, J. B.. Pathophysiology and treatment of Lassa fever. *Curr Top Microbiol Immunol.* 1987. 134:231-9

Fisher-Hoch, S.,McCormick, J. B.,Sasso, D.,Craven, R. B.. Hematologic dysfunction in Lassa fever. *J Med Virol.* 1988. 26:127-35

Fisher-Hoch, S.. Pathophysiology of shock and haemorrhage in viral haemorrhagic fevers. *Southeast Asian J Trop Med Public Health.* 1987. 18:390-1

Fleischer, K.,Kohler, B.,Kirchner, A.,Schmid, J.. [Lassa fever]. *Med Klin (Munich).* 2000. 95:340-5

Frame, J. D.. The story of Lassa fever. Part II: Learning more about the disease. *N Y State J Med.* 1992. 92:264-7

Gear, J. H.. Haemorrhagic fevers of Africa: an account of two recent outbreaks. *J S Afr Vet Assoc.* 1977. 48:5-8

Getso, K. I.,Balogun, M. S.,Gidado, S. O.,Oladejo, J.,Nasidi, A.,Nguku, P. M.,Akpan, H.,Ajisegiri, S.,Oyemakinde, A.. Lassa fever outbreak involving health care workers in Taraba state, Nigeria; march 2012. *American Journal of Tropical Medicine and Hygiene.* 2013. 1):132-133

Glover, S. C.. Dangerous pathogens: implications for staff and patients. *J Sterile Serv Manage.* 1983. 1:2-3

Godtfredsen, A.. [Lassa fever]. *Ugeskr Laeger.* 1977. 139:478-9

Grant, D. S.,Goba, A.,Shaffer, J. G.,Boisen, M. L.,Branco, L. M.,Ottomassathien, D.,Levy, D.,Bausch, D. G.,Garry, R. F.,Schieffelin, J. S.. Demographic and clinical risk factors for lassa fever in sierra Leone. *American Journal of Tropical Medicine and Hygiene.* 2012. 1):386

Hall, A. P.. Imported fever including malaria. *Practitioner.* 1982. 226:1521-31

Hamer, J. L.. Hoosiers abroad--report of a case of Lassa fever. *J Indiana State Med Assoc.* 1974. 67:659-60

Hamorrhagische Fieber - Bedrohung Fur Das Krankenhauspersonal?. *Hygiene + Medizin.* 1985. 10:419-423

Helmick, C. G.,Webb, P. A.,Scribner, C. L.,Krebs, J. W.,McCormick, J. B.. No evidence for increased risk of Lassa fever infection in hospital staff. *Lancet.* 1986. 2:1202-5

Hirabayashi, Y.,Oka, S.,Goto, H.,Shimada, K.,Kurata, T.,Fisher-Hoch, S. P.,McCormick, J. B.. [The first imported case of Lassa fever in Japan]. *Nihon Rinsho.* 1989. 47:71-5

Hirabayashi, Y.. [Lassa fever: its virological and clinical aspects]. *Ryoikibetsu Shokogun Shirizu.* 1999:80-4

Hotta, H.. [Viral hemorrhagic fever--Ebola hemorrhagic fever, Marburg disease and Lassa fever]. *Rinsho Byori.* 1998. 46:651-5

Hotton, J. M.,Bousquet, M.,Barry, B.,Lamour, O.. Aeromedical evacuation of patients with Lassa fever. *Aviat Space Environ Med.* 1991. 62:909-10

Ichikawa, S.,Ohya, H.,Ito, K.. [Introduction to sterilization and disinfection of medical wastes contaminated with human virus]. *Rinsho Byori.* 2000. Suppl 112:15-20

Inapogui, A. P.,Konstantinov, O. K.,Lapshov, V. N.,Comara, S. K.. [Characterization of contacts of the population of Guinea with synanthropic rodents as Lassa fever virus carriers]. *Med Parazitol (Mosk).* 2007. :47-51

Ishak, K. G.,Walker, D. H.,Coetzer, J. A.,Gardner, J. J.,Gorelkin, L.. Viral hemorrhagic fevers with hepatic involvement: pathologic aspects with clinical correlations. *Prog Liver Dis.* 1982. 7:495-515

Ivanov, A. P.,Rezapkin, G. V.,Dzagurova, T. K.,Tkachenko, E. A.. Indirect solid-phase immunosorbent assay for detection of arenavirus antigens and antibodies. *Acta Virol.* 1984. 28:240-5

Ivanov, A. P.,Tkachenko, E. A.,van der Groen, G.,Butenko, A. M.,Konstantinov, O. K.. [Indirect immunoenzyme method for the laboratory diagnosis of Lassa and Ebola hemorrhagic fevers]. *Vopr Virusol.* 1986. 31:186-90

Ivanov, A. P.,Tkachenko, E. A.,Van Der Groen, G.. Indirect enzyme-immunoassay for laboratory diagnosis of Lassa and Ebola hemorrhagic fevers. [Russian]. *Voprosy virusologii.* 1986. 31:186-190

Jerath, R.. Recent advances in viral zoonoses. *Int J Zoonoses.* 1979. 6:49-60

Knobloch, J.,McCormick, J. B.,Webb, P. A.,Dietrich, M.,Schumacher, H. H.,Dennis, E.. Clinical observations in 42 patients with Lassa fever. *Tropenmed Parasitol.* 1980. 31:389-98

Krasnianskii, V. P.,Potryvaeva, N. V.,Borisevich, I. V.,Gradoboev, V. N.,Pashanina, T. P.,Pshenichnov, V. A.. [An evaluation of the possibility of obtaining an inactivated vaccine against Lassa fever]. *Zh Mikrobiol Epidemiol Immunobiol.* 1994:74-5

La Fievre De Lassa. *Annales de l'Institut Pasteur/Actualites.* 1997. 8:223-231

La Fievre De Lassa. *Medecine et Armees.* 1977. 5:117-122

Lacy, M. D.,Smego, R. A.. Viral hemorrhagic fevers. *Adv Pediatr Infect Dis.* 1996. 12:21-53

Leber, W.,Hemmer, C.,Lafrenz, M.,Reisinger, E. C.. Tropical diseases - Microbiology. 2. Viral haemorrhagic fever. [German]

Leparc-Goffart, I.,Emonet, S. F.. [An update on Lassa virus]. *Med Trop (Mars).* 2011. 71:541-5

Les Nouvelles Fievres Virales Africaines: Ebola - Lassa - Marburg. *Revue de Medecine.* 1982. 23:1859-1864

Liao, B. S.,Byl, F. M.,Adour, K. K.. Audiometric comparison of Lassa fever hearing loss and idiopathic sudden hearing loss: evidence for viral cause. *Otolaryngol Head Neck Surg.* 1992. 106:226-9

Lukashevich, I. S.,Clegg, J. C.,Sidibe, K.. Lassa virus activity in Guinea: distribution of human antiviral antibody defined using enzyme-linked immunosorbent assay with recombinant antigen. *J Med Virol.* 1993. 40:210-7

Lukashevich, I. S.,Lukashevich, V. D.,Vladyko, A. S.,Diallo, M. S.,Ba, M. A.,Silla, A.. [Serological evidence of Lassa virus circulation in the Republic of Guinea]. *Vopr Virusol.* 1993. 38:24-8

Mahdy, M. S.,Chiang, W.,McLaughlin, B.,Derksen, K.,Truxton, B. H.,Neg, K.. Lassa fever: the first confirmed case imported into Canada. *Can Dis Wkly Rep.* 1989. 15:193-8

Mairuhu, A. T. A.,Brandjes, D. P. M.,Van Gorp, E. C. M.. Treating viral hemorrhagic fever. *IDrugs.* 2003. 6:1061-1066

Markin, V. A.,Markov, V. I.. [Viral haemorrhagic fevers--evolution of the epidemic potential]. *Zh Mikrobiol Epidemiol Immunobiol.* 2002:91-8

Matsubara, Y.,Seo, T.. [Lassa fever: epidemiology, symptomatology, management and prognosis]. *Nihon Rinsho.* 1980. 38:295-302

McCormick, J. B.,Fisher-Hoch, S. P.. Lassa fever. *Curr Top Microbiol Immunol.* 2002. 262:75-109

McCormick, J. B.. Epidemiology and control of Lassa fever. *Curr Top Microbiol Immunol.* 1987. 134:69-78

McCormick, J. B.. Lassa fever: epidemiology, therapy and vaccine development. *Kansenshogaku Zasshi.* 1988. 62 Suppl:353-66

Miasnenko, A. M.. [Lassa fever and Marburg disease]. *Voen Med Zh.* 1978. #volume#:42-6

Mohr, W.,Brinkmann, U.. [Lassa fever]. *Ergeb Inn Med Kinderheilkd.* 1978. 40:1-28

Mohr, W.,Brinkmann, U.. [Lassa fever]. *Internist (Berl).* 1975. 16:401-6

Mohr, W.,Brinkmann, U.. [Problems of clinical diagnosis and therapy of a new arena virus infection (Lassa fever)]. *Verh Dtsch Ges Inn Med.* 1975. 81:677-9

Mollaret, P.. [Latest viruses from virgin African forests, unknown factors in their epidemiology and the need for absolute isolation of all suspects. II. How to protect France?]. *Nouv Presse Med.* 1978. 7:3015-22

Mollaret, P.. [Latest viruses from virgin African forests, unknown factors in their epidemiology and the need for absolute isolation of all suspects]. *Nouv Presse Med.* 1978. 7:2919-20

Monat, T. P.. [Lassa fever, on-going problems and prospects]. *Vestn Akad Med Nauk SSSR.* 1977:35-42

Monath, T. P.. Lassa fever and Marburg virus disease. *WHO Chron.* 1974. 28:212-9

Monath, T. P.. Lassa fever. *Trop Doct.* 1973. 3:155-61

Monath, T. P.. Lassa fever: a new appraisal. *Niger Med J.* 1973. 3:162-3

Morikawa, S.. [Laboratory diagnosis of viral infections. 8. Viral hemorrhagic fever]. *Rinsho Byori.* 1998. Suppl 108:105-10

Morikawa, S.. [Lassa virus]. *Nihon Rinsho.* 2003. 61 Suppl 3:539-43

Murphy, F. A.. Control and eradication of exotic viruses affecting man. *Prog Med Virol.* 1979. 25:69-82

Nakounne, E.,Selekon, B.,Morvan, J.. [Microbiological surveillance: viral hemorrhagic fever in Central African Republic: current serological data in man]. *Bull Soc Pathol Exot.* 2000. 93:340-7

Negredo Anton, A. I.,de Ory Manchon, F.,Sanchez-Seco Farinas, M. P.,Franco Narvaez, L.,Gegundez Camara, M. I.,Navarro Mari, J. M.,Tenorio Matanzo, A.. [Microbiological diagnosis of emerging arboviral and rodent borne diseases]. *Enferm Infecc Microbiol Clin.* 2015. 33:197-205

Nielsen, G.. [Lassa virus disease]. *Dtsch Med Wochenschr.* 1974. 99:1319-21

Nuti, M.. [Lassa fever]. *Minerva Med.* 1974. 65:3875-80

O'Hearn, A.,Voorhees, M.,Ames, A.,Fair, J.,Schoepp, R.. Development of advanced sero-assays to broaden diagnostic and surveillance capability in West Africa. *American Journal of Tropical Medicine and Hygiene.* 2014. 1):28

Owen, J.. [Lassa fever surveillance: the need for better communication]. *Can J Public Health.* 1977. 68:101-5

Pautov, V. N.. [Lassa fever (review of the literature)]. *Zh Mikrobiol Epidemiol Immunobiol.* 1973. 50:24-5

Picardi, A.,Gentilucci, U. V.,Zardi, E. M.,D'Avola, D.,Amoroso, A.,Afeltra, A.. The role of ribavirin in the combination therapy of hepatitis C virus infection. *Curr Pharm Des.* 2004. 10:2081-92

Pigott, D. C.. Hemorrhagic fever viruses. *Crit Care Clin.* 2005. 21:765-83, vii

Pokrovskii, V. V.. [New African hemorrhagic fevers]. *Ter Arkh.* 1979. 51:119-23

Pshenichnov, V. A.,Makhlai, A. A.,Mikhailov, V. V.. [Research with the Marburg, Lassa and Ebola viruses]. *Vopr Virusol.* 1993. 38:54-8

Raeburn, P.. Lassa fever. *Nurs Times.* 1976. 72:37-8

Rodrigues, F. M.,Gupta, N. P.,Pinto, B. D.. Serological survey for the detection of antibodies to lassa virus in India. *J Indian Med Assoc.* 1978. 70:25-8

Ryan, H.. Infectious diseases. Moving an infectious patient by air. *Nursing (Lond).* 1990. 4:24-6

Saijo, M.. [Clinical aspects of viral hemorrhagic fever]. *Nihon Rinsho.* 2005. 63:2161-6

Saijo, M.. [Lassa fever]. *Nihon Rinsho.* 2007. 65 Suppl 3:40-3

Sarrat, H.,Camain, R.,Baum, J.,Robin, Y.. [Histopathological diagnosis of hepatitis due to Lassa virus]. *Bull Soc Pathol Exot Filiales.* 1972. 65:642-50

Saxe, S. E.,Gardner, P.. The returning traveler with fever. *Infect Dis Clin North Am.* 1992. 6:427-39

Schlaeffer, F.,Bar-Lavie, Y.,Sikuler, E.,Alkan, M.,Keynan, A.. Evidence against high contagiousness of Lassa fever. *Trans R Soc Trop Med Hyg.* 1988. 82:311

Shlaeffer, F.,Sikuler, E.,Keynan, A.. [Lassa fever--first case diagnosed in Israel]. *Harefuah.* 1988. 114:12-4

Simmons, F. B.. Lassa fever and sudden hearing loss. *Otolaryngol Head Neck Surg.* 1993. 109:559

Simpson, D. I.. [Viral hemorrhagic fevers in man]. *Bull World Health Organ.* 1979. 57:19-32

Snell, N.. Ribavirin therapy for lassa fever. *Practitioner.* 1988. 232:432

Solbrig, M. V.. Lassa fever and central nervous system diseases: A review. *Neurological Infections and Epidemology.* 1997. 2:13-18

Swaan, C. M.,van den Broek, P. J.,Wijnands, S.,van Steenbergen, J. E.. Management of viral haemorrhagic fever in the Netherlands. *Euro Surveill.* 2002. 7:48-50

Tani, H.,Fukushi, S.,Yoshikawa, T.,Saijo, M.,Morikawa, S.. [Arenavirus infections]. *Uirusu.* 2012. 62:229-38

Taniguchi, K.. [Viral hemorrhagic fevers--Ebola hemorrhagic fever, Marburg virus disease, and Lassa fever]. *Nihon Naika Gakkai Zasshi.* 2004. 93:2303-8

Ter Meulen, J.,Lukashevich, I.,Sidibe, K.,Inapogui, A.,Marx, M.,Dorlemann, A.,Yansane, M. L.,Koulemou, K.,Chang-Claude, J.,Schmitz, H.. Hunting of peridomestic rodents and consumption of their meat as possible risk factors for rodent-to-human transmission of Lassa virus in the Republic of Guinea. *Am J Trop Med Hyg.* 1996. 55:661-6

ter Meulen, J.. Lassa fever: immuno-epidemiological approach to the study of an endemic viral haemorrhagic fever. *Med Trop (Mars).* 2000. 60:20-3

The threat of emerging infections. *Glob Issues.* 1996. 1:31-4

Thomas, J.,Goasguen, J.,Gautier, D.. Lassa fever. [French]

Tobin, E. A.,Asogun, D. A.,Ehidiamen, G.,Elugbe, B.,Osiemi, B.. Engaging Market Traders in Lassa Fever Campaign: Assessment of Knowledge and Risk Behaviour. *West Afr J Med.* 2015. 34:89-93

Tropenkrankheiten - Mikrobiologie. 2. Virales hamorrhagisches fieber. *Internistische Praxis.* 2002. 42:57-62

van der Heide, R. M.. [A patient with Lassa fever from the Upper Volta, diagnosed in the Netherlands]. *Ned Tijdschr Geneeskd.* 1982. 126:566-9

Van der Waals, F. W.,Pomeroy, K. L.,Goudsmit, J.,Asher, D. M.,Gajdusek, D. C.. Hemorrhagic fever virus infections in an isolated rainforest area of central Liberia. Limitations of the indirect immunofluorescence slide test for antibody screening in Africa. *Trop Geogr Med.* 1986. 38:209-14

Veldkamp, P. J.,Schippers, E. F.. [A man with fatal Lassa fever following a stay in Sierra Leone]. *Ned Tijdschr Geneeskd.* 2002. 146:2201-4

Vella, E. E.. Lassa fever (LF) and Marburg disease (MVD): occurrences, origins and diagnoses. *R Soc Health J.* 1978. 98:150-2

Visser, L. G.,Schippers, E. F.,Swaan, C. M.,van den Broek, P. J.. [How to treat a patient with indications for an infectious viral hemorrhagic fever]. *Ned Tijdschr Geneeskd.* 2002. 146:2183-8

Vladyko, A. S.,Zaitseva, V. N.,Trofimov, N. M.,Shkolina, T. V.,Scheslenok, E. P.,Boshchenko Iu, A.,Petkevich, A. S.. [False-positive reactions in laboratory diagnosis of Lassa, Marburg, and Ebola viral hemorrhagic fevers and AIDS]. *Vopr Virusol.* 1997. 42:66-70

Vuksanovic, P.. [Lassa-fever a new dangerous virus infection]. *Med Arh.* 1974. 28:575-7

Walls, B.. Lassa fever and pregnancy. *Midwives Chron.* 1985. 1168:136-8

Walters, A.,Pilkington, D. B.. Radiography in a secure isolation unit. *Radiography.* 1984. 50:11-3

Weise, H. J.. ["New" infectious diseases and their prevention]. *MMW Munch Med Wochenschr.* 1978. 120:1559-60

Winn, W. C., Jr.,Monath, T. P.,Murphy, F. A.,Whitfield, S. G.. Lassa virus hepatitis. Observations on a fatal case from the 1972 Sierra Leone epidemic. *Arch Pathol.* 1975. 99:599-604

Wone, I.,de Lauture, H.. [Virological and clinical notes on recent viral epidemics in Zaire]. *Dakar Med.* 1979. 24:56-9

Wood, J.. Exotic diseases--communicable, dangerous, and, yes, possible!. *Can Nurse.* 1982. 78:18-22

Yamaguchi, T.,Tsuji, M.,Imagawa, Y.. [A first Lassa fever from Sierra Leone treated in high security ward]. *Nihon Rinsho.* 1989. 47:76-81
Yanase, O.,Motomiya, T.,Watanabe, K.,Tokuyasu, Y.,Sakurada, H.,Tejima, T.,Hiyoshi, Y.,Sugiura, M.,Yabata, Y.,Kitazumi, H.. [Lassa fever associated with effusive constrictive pericarditis and bilateral atrioventricular annular constriction: a case report]. *J Cardiol.* 1989. 19:1147-56
Zuckerman, A. J.,Simpson, D. I.. Exotic virus infections of the liver. *Prog Liver Dis.* 1979. 6:425-38
