# Supplementary material for: A rapid research needs appraisal methodology to identify evidence gaps to inform clinical research priorities in response to outbreaks—results from the Lassa fever pilot
Source: BMC Med. 2019 Jun 11;17:107. doi: 10.1186/s12916-019-1338-1 (PMC6560772; doi:10.1186/s12916-019-1338-1)
Supplement: Supplementary file 3 — Articles for which data were not extracted. (PDF 163 kb) [file 12916_2019_1338_MOESM3_ESM.pdf]

Additional file 3. Articles for which data were not extracted

The tables below provide bibliographies of articles included but not data extracted.

Not extracted due to time-constraints (n=14)

Bibliography	Year	Study design	Domains
Bausch, D. G., Lassa fever in Guinea: I. Epidemiology of human disease and clinical observations. Vector Borne Zoonotic Dis. 2001. 1:269-81	2001	Cohort	CP/NH
Bausch, D. G.,Rollin, P. E, et al. Diagnosis and clinical virology of Lassa fever as evaluated by enzyme-linked immunosorbent assay, indirect fluorescent-antibody test, and virus isolation. J Clin Microbiol. 2000. 38:2670-7	2000	Not identified	Not identified
Bonney, J. H. K.,Nyarko, E. O., et al. Molecular confirmation of Lassa fever imported into Ghana. African Journal of Laboratory Medicine. 2016. 5 (1)	2016	Not identified	Not identified
Ehichioya, D.,Asogun, D.,et al. A retrospective laboratory analysis of clinically diagnosed Lassa fever cases in a tertiary hospital in Nigeria. International Journal of Infectious Diseases. 2010. 14:e209-e210	2010	Not identified	Not identified
Emmerich, P.,Gunther, S.,Schmitz, H. Strain-specific antibody response to Lassa virus in the local population of west Africa. J Clin Virol. 2008. 42:40-4	2008	Not identified	Not identified
Emmerich, P.,Thome-Bolduan, et al. Reverse ELISA for IgG and IgM antibodies to detect Lassa virus infections in Africa. J Clin Virol. 2006. 37:277-81	2006	Not identified	Not identified
Frame, J. D.,. The use of Lassa fever convalescent plasma in Nigeria. Trans R Soc Trop Med Hyg. 1984. 78:319-24	1984	Cohort	DR
Fraser, D. Lassa fever in the Eastern Province of Sierra Leone, 1970-1972. I. Epidemiologic studies. Am J Trop Med Hyg. 1974. 23:1131-9	1974	Cohort	CP/NH and T
McCormick, J. B.,Lassa virus hepatitis: a study of fatal Lassa fever in humans. Am J Trop Med Hyg. 1986. 35:401-7	1986	Cohort	CP/NH
Olschlager, S.,Lelke, M., et al. Improved detection of Lassa virus by reverse transcription-PCR targeting the 5' region of S RNA. J Clin Microbiol. 2010. 48:2009-13	2010	Not identified	Not identified
Shaffer, J. G.,Grant, D. S., et al. Lassa fever in post-conflict Sierra Leone. PLoS Negl Trop Dis. 2014. 8:e2748	2014	Not identified	Not identified
Ter Meulen, J., Detection of Lassa virus anti nucleoprotein immunoglobulin G (IgG) and IgM antibodies by a simple recombinant immunoblot assay for field use. J Clin Microbiol. 1998. 36:3143-8	1998	Cohort	D
Tobin, E. A.,Asogun, D.,et al.. Risk factors for Lassa fever in endemic communities of Edo State, Nigeria. International Journal of Infectious Diseases. 2014. 21:258-259	2014	Not identified	Not identified
Webb, P. A., Lassa fever in children in Sierra Leone, West Africa. Trans R Soc Trop Med Hyg. 1986. 80:577-82	1986	Cohort	CP/NH, I

CP/NH: Clinical phenotype and natural history of disease, D: Diagnostics, DR: Drug therapy, I: Immune response, T: Transmission

Case reports and case series (n=58)

Bibliography	Year	Study design	Domains
Ajayi, N. A.,Lassa fever - full recovery without ribavarin treatment: a case report. Afr Health Sci. 2014. 14:1074-7	2014	Case report	CP/NH, I
Akpede, G. O.,Manifestations and outcomes of lassa fever in nigerian children: A case series. Archives of Disease in Childhood. 2012. 97:A38-A39	2012	Case series	CP/NH
Amorosa, V.Imported Lassa fever, Pennsylvania, USA, 2010. Emerg Infect Dis. 2010. 16:1598-600	2010	Case report	CP/NH, S
Anonymous,. Imported Lassa fever--New Jersey, 2004. Mmwr. 2004. Morbidity and mortality weekly report. 53:894-897	2004	Case report	CP/NH
Anonymous. Lassa fever 1982. Br Med J (Clin Res Ed). 1983. 287:48	1983	Case report	CP/NH
BalasegaraExamples of tropical disease control in the humanitarian medical programmes of MSF and Merlin. Trans R Soc Trop Med Hyg. 2006. 100:327-34	2006	Case report	T
Bello, O. O.,A Lassa Fever in Pregnancy: Report of 2 Cases Seen at the University College Hospital, Ibadan. Case Rep Obstet Gynecol. 2016. 2016:9673683	2016	Case series	CP/NH, S

Bond, N.,A historical look at the first reported cases of Lassa fever: IgG antibodies 40 years after acute infection. Am J Trop Med Hyg. 2013. 88:241-4	2013	Case series	I
Bowen, G. S., Lassa fever in Onitsha, East Central State, Nigeria in 1974. Bull World Health Organ. 1975. 52:599-604	1975	Case series	CP/NH
Branco, L. M., Lassa hemorrhagic fever in a late term pregnancy from northern Sierra Leone with a positive maternal outcome: case report. Virol J. 2011. 8:404	2011	Case report	CP/NH, T, D, I, DR
Carey, D. E.,Lassa fever. Epidemiological aspects of the 1970 epidemic, Jos, Nigeria. Trans R Soc Trop Med Hyg. 1972. 66:402-8	1972	Case series	CP/NH, I
Clayton, A. J.. Lassa immune serum. Bull World Health Organ. 1977. 55:435-9	1977	Case series	S
Cooper, C. B.,A case of Lassa fever: experience at St Thomas's Hospital. Br Med J (Clin Res Ed). 1982. 285:1003-5	1982	Case report	CP/NH, T, I
Cummins, D., Exchange transfusion of a patient with fulminant Lassa fever. Postgrad Med J. 1991. 67:193-4	1991	Case report	S
Dongo, A. E.,Lassa fever presenting as acute abdomen: a case series. Virol J. 2013. 10:123	2013	Case series	CP, I, DR
Dzotsi, E. K., The first cases of Lassa fever in Ghana. Ghana Med J. 2012. 46:166-70	2012	Case series	CP/NH, S
Edington, G. M.,The pathology of Lassa fever. Trans R Soc Trop Med Hyg. 1972. 66:381-9	1972	Case series	CP/NH
Edo, A. E.,Hyperglycemic crisis precipitated by Lassa fever in a patient with previously undiagnosed type 2 diabetes mellitus. Niger J Clin Pract. 2014. 17:658-61	2014	Case report	CP/NH, DR, S
Emond, R. T., A case of Lassa fever: clinical and virological findings. Br Med J (Clin Res Ed). 1982. 285:1001-2	1982	Case report	CP/NH, T, I
Emond, R. T.,Managing Lassa fever. Lancet. 1984. 2:926	1984	Case report	CP/NH
Fabiyi, A., Use of the complement fixation (CF) test in Lassa fever surveillance. Evidence for persistent CF antibodies. Bull World Health Organ. 1975. 52:605-8	1975	Case series	I
Fisher-Hoch, S. Safe intensive-care management of a severe case of Lassa fever with simple barrier nursing techniques. Lancet. 1985. 2:1227-9	1985	Case report	CP/NH
Frame, J. D.,Lassa fever, a new virus disease of man from West Africa. I. Clinical description and pathological findings. Am J Trop Med Hyg. 1970. 19:670-6	1970	Case series	CP/NH
Frame, J. D.. Lassa fever, a new virus disease of man from west africa. I. Clinical description and pathological findings. Amer.J.Trop.Med.Hyg. 1970. 19:339-350	1970	Case series	CP/NH
Gilles, H. M., Lassa fever: retrospective diagnosis of two patients seen in Great Britain in 1971. Br Med J. 1976. 2:1173	1976	Case series	T
Grahn, A.,, P.,Imported Case of Lassa Fever in Sweden With Encephalopathy and Sensorineural Hearing Deficit. Open Forum Infect Dis. 2016. 3:ofw198	2016	Case report	CP/NH, I
Grove, J. N.Capacity building permitting comprehensive monitoring of a severe case of Lassa hemorrhagic fever in Sierra Leone with a positive outcome: case report. Virol J. 2011. 8:314	2011	Case report	CP/NH, T, P, D, DR
Grundy, D. J., Isolated case of Lassa fever in Zaria, Northern Nigeria. Lancet. 1980. 2:649-50	1980	Case report	CP/NH, S
Gunther, S.,Lassa fever encephalopathy: Lassa virus in cerebrospinal fluid but not in serum. J Infect Dis. 2001. 184:345-9	2001	Case report	CP/NH, D
Hirabayashi, Y., An imported case of Lassa fever with late appearance of polyserositis. J Infect Dis. 1988. 158:872-5	1988	Case report	CP/NH
Holmes, G. P.,. Lassa fever in the United States. Investigation of a case and new guidelines for management. N Engl J Med. 1990. 323:1120-3	1990	Case report	CP/NH, S
Jahrling, P. Early diagnosis of human Lassa fever by ELISA detection of antigen and antibody. Lancet. 1985. 1:250-2	1985	Case series	I
Kimura, M., Diagnosis of febrile illnesses in returned travelers using the PC software GIDEON. Travel Med Infect Dis. 2005. 3:157-60	2005	Case report	D
Kitching, A., A fatal case of Lassa fever in London, January 2009. Euro Surveill. 2009. 14	2009	Case report	CP/NH, D
Kyei, N. N., Imported Lassa fever: a report of 2 cases in Ghana. BMC Infect Dis. 2015. 15:217	-	Case series	CP/NH, DR, S
Lamb, D.. Evaluation of infection control practices during an AE. Br J Nurs. 2006. 15:543-7	2006	Case report	CP/NH, DR
Lassa fever, case imported to Germany. Wkly Epidemiol Rec. 2000. 75:17-8	2000	Case report	CP/NH, T

Leifer, E., Lassa fever, a new virus disease of man from West Africa. II. Report of a laboratory-acquired infection treated with plasma from a person recently recovered from the disease. Am J Trop Med Hyg. 1970. 19:677-9	1970	Case report	CP/NH, DR
Lloyd, G. Identification of Lassa fever virus infection with recombinant nucleocapsid protein antigen. Lancet. 1989. 2:1222	1989	Case report	CP/NH, I, DR
Lotz, E., Aeromedical evacuation using an aircraft transit isolator of a patient with Lassa fever. Aviat Space Environ Med. 2012. 83:527-30	2012	Case report	CP/NH, T, I, DR
Macher, A. M.,Historical Lassa fever reports and 30-year clinical update. Emerg Infect Dis. 2006. 12:835-7	2006	Case series	D
Mertens, P. E.,Clinical presentation of Lassa fever cases during the hospital epidemic at Zorzor, Liberia, March-April 1972. Am J Trop Med Hyg. 1973. 22:780-4	1973	Case series	CP/NH, T
Monath, T. P.,Lassa fever in the Eastern Province of Sierra Leone, 1970-1972. II. Clinical observations and virological studies on selected hospital cases. Am J Trop Med Hyg. 1974. 23:1140-9	1974	Case series	CP/NH, S
Monson, M. H.,Pediatric Lassa fever: a review of 33 Liberian cases. Am J Trop Med Hyg. 1987. 36:408-15	1987	Case series	CP/NH
Ogunniyi, A. O., Lassa fever outbreak investigation in a Nigerian bakery - August, 2012. International Journal of Infectious Diseases. 2014. 21:400	2014	Case series	CP
Okogbenin, S. New lessons from a case series review of Lassa fever in pregnancy. International Journal of Infectious Diseases. 2010. 14:e380	2010	Case series	CP/NH, DR
Okokhere, P. O.,ISensorineural hearing loss in Lassa fever: two case reports. J Med Case Rep. 2009. 3:36	2009	Case report	CP/NH, I, DR
Okokhere,Aseptic Meningitis Caused by Lassa Virus: Case Series Report. Case Rep Neurol Med. 2016. 2016:1978461	2016	Case series	CP, DR
Oppenheimer, L.. A case of Lassa fever. Br Med J (Clin Res Ed). 1982. 285:1576-7	1982	Case series	CP/NH
Schmitz, H.,Monitoring of clinical and laboratory data in two cases of imported Lassa fever. Microbes Infect. 2002. 4:43-50	2002	Case report	CP/NH, DR, S
Sharp, P. C.. Lassa fever in children. J Infect. 1982. 4:73-7	1982	Case series	CP/NH
Swaan, C. M.,Management of a patient with Lassa fever to prevent transmission. J Hosp Infect. 2003. 55:234-5	2003	Case report	CP/NH, DR
ter Meulen, J., Short communication: Lassa fever in Sierra Leone: UN peacekeepers are at risk. Trop Med Int Health. 2001. 6:83-4	2001	Case report	CP/NH, T, S
Troup, J. M., An outbreak of Lassa fever on the Jos plateau, Nigeria, in January-February 1970. A preliminary report. Am J Trop Med Hyg. 1970. 19:695-6	1970	Case series	CP/NH, T
Walker, D. H.,Pathologic and virologic study of fatal Lassa fever in man. Am J Pathol. 1982. 107:349-56	1982	Case report	CP/NH, I
White, H. A.. Lassa fever. A study of 23 hospital cases. Trans R Soc Trop Med Hyg. 1972. 66:390-401	1972	Case series	CP/NH
Woodruff, A. W.,Lassa fever in Britain: an imported case. Br Med J. 1973. 3:616-7	1973	Case report	CP/NH
Wulff, H. Immunoglobulin M and G responses measured by immunofluorescence in patients with Lassa or Marburg virus infections. Bull World Health Organ. 1979. 57:631-5	1979	Case series	I

CP/NH: Clinical phenotype and natural history of disease, D: Diagnostics, DR: Drug therapy, I: Immune response, S; Supportive care, T: Transmission
