# Supplementary material for: A rapid research needs appraisal methodology to identify evidence gaps to inform clinical research priorities in response to outbreaks—results from the Lassa fever pilot
Source: BMC Med. 2019 Jun 11;17:107. doi: 10.1186/s12916-019-1338-1 (PMC6560772; doi:10.1186/s12916-019-1338-1)
Supplement: Supplementary file 4 — Data extraction summary overview. (PDF 191 kb) [file 12916_2019_1338_MOESM4_ESM.pdf]

Additional file 4. Data extraction summary overview

Bibliography	Study design	Study objectives	Number of participants	Population demographics	Intervention	Comparator	Description of outcomes
Ajayi, N. A., Containing a Lassa fever epidemic in a resource-limited setting: outbreak description and lessons learned from Abakaliki, Nigeria (January-March 2012). Int J Infect Dis. 2013. 17:e1011-6	Cross-sectional	Reporting response to and outcomes of LF outbreak, including transmission to health workers.	Tot:21 (1 index case), 110 contacts	20 suspected and confirmed cases: 14 men, 6 women, age median 36, range 12-47.	Ribavirin		Diagnosis<br>Mortality
Akhuemokhan, O. C.,Lassa fever and convulsions associated with fever: A case-control study. Archives of Disease in Childhood. 2017. 102:A122	Case-control	To determine the prevalence of LF in children with fever, compare seizure characteristics, clinical features and outcomes.	Tot: 373 children (with fever of >38 C), n=108 febrile with convulsions, n= 265 febrile with no convulsions	Not reported		Febrile children without convulsions (controls)	Prevalence
Akhuemokhan, O. C.Prevalence of Lassa Virus Disease (LVD) in Nigerian children with fever or fever and convulsions in an endemic area. PLoS Negl Trop Dis. 2017. 11:e0005711	Case-control	The aim of this study was to determine the prevalence and presentation of Lassa virus disease (LVD) in febrile children with and without convulsions.	Tot: 373 children with fever<br>108 cases (61 with febrile convulsions, 47 with non-febrile convulsions)<br>265 controls	58.5% male, 76.7% ≤5 years			Prevalence<br>Case fatality rate
Akpede, G.,Prevalence and presentation of Lassa fever in Nigerian children. International Journal of Infectious Diseases. 2010. 14:e380	Cohort	To report the prevalence, presentation and outcome in Nigerian children (in a single institution).	Tot:22	n=22 age range <2 yrs - > 5 years old, 13 Male , 9 Female			Symptoms
Asogun, D. A.Molecular diagnostics for lassa fever at Irrua specialist teaching hospital, Nigeria: lessons learnt from two years of laboratory operation. PLoS Negl Trop Dis. 2012. 6:e1839	Cohort	To describe the establishment of a diagnostic service for Lassa fever and analyze the data recorded during two years of operation.	Tot: 198	Median age 32 (IQR 23-46) 14 children 0-4 years, 9 children 5-9 years, 7 children 10-15 years	Ribavirin		Case fatality rate<br>Length of stay<br>Signs and symptoms
Asogun, D., Lassa fever practice challenges in Nigeria. International Journal of Infectious Diseases. 2012. 16:e69	Cohort	To review records and describe characteristics and outcomes of LF cases in a single hospital.	No information	Not reported			Case fatality rate
Atkin, S., The first case of Lassa fever imported from Mali to the United Kingdom, February 2009. Euro Surveill. 2009. 14	Cohort	Reporting contact tracing and monitoring after a fatal LF index case was imported to London from Mali.	Tot: 167 contacts	Not reported			Human-to-human transmission
Branco, L. M.,Emerging trends in Lassa fever: redefining the role of immunoglobulin M and inflammation in diagnosing acute infection. Virol J. 2011. 8:478	Cohort	Assessing the importance of different diagnostic tests in prognosis. Comparison of serology in infected, suspected and normal samples.	Tot: 1909 suspected LF cases. 456 tested LASV Ag and IgM	Insufficent data. Majority of patients female, majority >40 years old		Mortality outcome compared between diagnostic groups:<br>Ag+ IgM+<br>Ag+ IgM-<br>Ag- IgM+<br>Ag- IgM-	Mortality<br>Serology

						Serology compared between Lassa, non-Lassa febrile patients, healthy controls.	
Brown, B. L., Suspected lassa fever (LF) case outcomes: A comparison to a non-febrile population in Sierra Leone. Clinical Chemistry. 2014. 1):S38	Case-control	To evaluate the difference in clinical and laboratory outcomes between LF cases and non-febrile controls.	Tot: 175. 57 cases, 118 controls	Not reported		Controls: non-febrile Sierra Leoneans with a temperature of ≤37.5°C.	Biochemical laboratory parameters
Crowcroft, N. S.,The public health response to a case of Lassa fever in London in 2000. J Infect. 2004. 48:221-8	Cohort	To assess public health response to imported case of LF.	Tot:125 contacts	Not reported			Human-to-human transmission
Cummins. Acute sensorineural deafness in Lassa fever. JAMA. 1990. 264:2093-6	Cohort	To compare incidence of sensorineural hearing loss (SNHL) in Lassa fever and non-Lassa febrile patients on and following discharge.	Cohort, Total: 69, n= 49 LF cases<br>Cross-sectional study: Total: 96, Case-control study:<br>Total = 64	Prospective cohort:<br>LF cases: 18 men, 31 women, mean age 30.2 (SD 10.1). Non-LF controls:<br>10 men, 10 women, 30.1 (SD 10.7)<br>Case-control study: Cases: 15 men, 17 women, mean age 41.7 (SD 13.2).<br>Controls: age and sex matched			Sensorineural hearing loss
Dahmane, A. Constraints in the diagnosis and treatment of Lassa Fever and the effect on mortality in hospitalized children and women with obstetric conditions in a rural district hospital in Sierra Leone. Trans R Soc Trop Med Hyg. 2014. 108:126-32	Cross-sectional	To assess treatment and outcomes of LF cases in paediatric and obstetric wards.	Tot: 84 suspected LF cases, n= 36 laboratory confirmed	84 patients with suspected LF; 62 (73%) were children aged l< five years, n=38 (45%) were females.<br>Among 36 confirmed cases, there were 24 (67%) children aged < five years; n=16 (44%) were female	Ribavirin		Administration of ribavirin<br>Mortality
Demby, A. H.,Early diagnosis of Lassa fever by reverse transcription-PCR. J Clin Microbiol. 1994. 32:2898-903	Cross-sectional	Validation of RT-PCR for diagnosis of LF using serum samples taken on admission from patients with fever and other signs and symptoms consistent with LF.	Tot: 188, n= 29 with LF, 32 without LF, n= 127 healthy blood donors	Not reported	RT-PCR<br>IFA	Immunofluorescence assay (IFA) for diagnosis of LF.	Diagnostic methods
Ehichioya, Hospital-based surveillance for Lassa fever in Edo State, Nigeria, 2005-2008. Trop Med Int Health. 2012. 17:1001-4	Cohort	To estimate the burden of Lassa fever in northern and central Edo,Nigeria.	Tot: 511, Part I: 60 inpatients (25 with LF), Part II: 451 outpatients (8 with Lassa fever)	Not reported	Ribavirin		Symptoms<br>Drug therapy effect Case fatality rate
Fisher-Hoch, S. P.,Unexpected adverse reactions during a clinical trial in rural west Africa. Antiviral Res. 1992. 19:139-47	Cross-sectional	To assess incidence of rigours as an adverse reaction to Ribavirin in LF patients.	Study 1: Tot: 93 LF Patients<br>Study 2: Tot: 2117 injections	Not reported	Ribavirin		Adverse reaction to Ribavirin

Fisher-Hoch. Review of cases of nosocomial Lassa fever in Nigeria: the high price of poor medical practice. BMJ. 1995. 311:857-9	Cohort	To investigate two hospital outbreaks of Lassa fever in southern central Nigeria.	Tot: 34 patients with LF	20 patients, 9 healthcare workers, 1 family contact			Case-fatality rate<br>Nosocomial transmission
Frame, J. D. Clinical features of Lassa fever in Liberia. Rev Infect Dis. 1989. 11 Suppl 4:S783-9	Cohort	This report deals with clinical Lassa fever in Liberia.	Tot: 246 patients	125 not pregnant, 32 pregnant<br>7 postpartum, 67 men<br>15 children <12 years			Case-fatality rate<br>Platelet counts Symptoms
Getso, K. I. Lassa fever outbreak involving healthcare workers in Taraba State, Nigeria, March 2012. International Journal of Infectious Diseases. 2014. 21:216	Cohort	Investigate a reported outbreak of Lassa fever in Taraba State, Nigeria to confirm the outbreak, determine its extent, characterize the outbreak and institute public health actions.	Tot:35 cases	Females 51%, Age group 25-34 years 40%			Case fatality rate<br>Symptoms
Haas, W. H., Imported Lassa fever in Germany: surveillance and management of contact persons. Clin Infect Dis. 2003. 36:1254-8	Cohort	Reports the epidemiological and virological investigation of 157 contact persons with different levels of exposure to a symptomatic patient with Lassa fever in Germany.	Tot: 232 contacts, n= 149 tested serologically	Not reported	Ribavirin (PEP)		Human-to-human transmission Ribavirin PEP
Hadi, C. M., Ribavirin for Lassa fever postexposure prophylaxis. Emerg Infect Dis. 2010. 16:2009-11	Cohort	to examine adherence and adverse effects associated with oral ribavirin given as PEP during an outbreak.	Tot: 23 (92%) of 25 persons known to have been prescribed ribavirin PEP	mean age 38 years (range 23–73 years); 14 (61%) male	Ribavirin (PEP)		Human-to-human transmission<br>Ribavirin PEP
Ibekwe, T. S., Early-onset sensorineural hearing loss in Lassa fever. Eur Arch Otorhinolaryngol. 2011. 268:197-201	Non-randomized controlled study	To investigate the incidence and features of sensorineural hearing loss in the acute phase of LF.	Tot: n=74 (n= 37 Cases, n= 37 Controls)	Cases: mean age of 35.3 § 14.1 (range 11–61 years). 16 (43.2%) males, 21 (56.8%) females.<br>Controls: mean age of 37.0 § 14.5 (range 17–59 years) males 13 (35.1%) and females 24 (64.9).		Cases (acute LF) compared with controls (non-Lassa febrile patients).	Sensourineural hearing loss (SNHL)<br>Case fatality rate
Ibekwe, T. S., The sensitivity and specificity of Lassa virus IgM by ELISA as screening tool at early phase of Lassa fever infection. Niger Med J. 2012. 53:196-9	Case-control	To investigate the adequacy of Lassa virus-specific IgM antibody as an early marker in LF diagnosis in our environment.	Tot: 72<br>Cases: 37<br>Controls: 35	19 (54.3%) males, 16 (45.7%) females. Mean age 35±12.8 years (range 24-48 years	IgM assay	Gold standard: LAV--specific RT–PCR for LF antigen<br>The PCR used was based on the primers S36+ and LVS 339.	IgM assay for diagnostics
Inegbenebor. Prevention of lassa Fever in Nigeria. Trans R Soc Trop Med Hyg. 2010. 104:51-4	Cohort	To determine the case-fatality rate of Lassa fever in Irrua Specialist Teaching Hospital.	Tot: 64 patients with LF	48 male, 16 female	Ribavirin		Case-fatality rate<br>Drug therapy effect
Iroezindu, M. O.,Lessons learnt from the management of a case of Lassa fever and follow-up of nosocomial primary contacts in Nigeria during Ebola virus disease outbreak in West Africa. Trop Med Int Health. 2015. 20:1424-1430	Cohort	To trace primary contacts of fatal index LF case, assess possible transmission and effectiveness of Ribavirin prophylaxis.	Tot:121, primary contacts, hospital staff, inpatients	mean age 32.8 (SD 6.6 years)<br>65.3% were women.	Ribavirin (PEP)		Human-to-human nosocomial transmission Ribavirin PEP<br>Adverse reactions

Isa, S. E.,Postexposure prophylaxis for Lassa fever: Experience from a recent outbreak in Nigeria. Niger Med J. 2016. 57:246-50	Cohort	To investigate the role of oral ribavirin postexposure prophylaxis (orPEP) in preventing LF among the primary contacts of confirmed cases.	66 (N=37Any exposure / contact - High Risk exposures - N= 29) Oral Ribavirin - N= 21	mean age 40.1 years (±10.9). 18 (48.6%) male.	Ribavirin (PEP)		Human-to-human nosocomial transmission<br>Ribavirin
Johnson, K. M. Clinical virology of Lassa fever in hospitalized patients. J Infect Dis. 1987. 155:456-64	Cohort	To determine association between levels of viraemia and other clinical manifestations and outcomes of LF.	392 LF cases amongst 648 febrile patients. Tot: 137 LF cases	No information			Survival by levels of viremia and AT
Keane, E., Lassa fever in Panguma Hospital, Sierra Leone, 1973-6. Br Med J. 1977. 1:1399-402	Cross-sectional	To asses endemic staus and outcomes of Lassa fever at the sites.	Panguma: Tot:156 suspected LF cases (40 serologically confirmed)<br>Segbwema: Tot:108 suspected LF cases (11 serologically confirmed)	Panama: 92 men, 55 women (inconsistent with total of 156)<br>Segbwema: 36 men, 60 women, 12 children			Mortality<br>Human-to-human nosocomial transmission
Kerneis, S., Prevalence and risk factors of Lassa seropositivity in inhabitants of the forest region of Guinea: a cross-sectional study. PLoS Negl Trop Dis. 2009. 3:e548	Cross-sectional	(i) estimate the prevalence of Lassa virus–specific IgG antibodies (LV IgG) in the human population of a rural area of Guinea<br>(ii) identify risk factors for positive LV IgG.	Tot:1424 subjects interviewed<br>1400 serum samples collected- 977 sera tested for LF	792/1424 (56%) women.<br>Median age 30 IR [2–18–46–99]			Prevalence of seropositivity<br>Risk exposures
Li, W. G.,The etiology of Ebola virus disease-like illnesses in Ebola virusnegative patients from Sierra Leone. Oncotarget. 2016. 7:27910-5	Cross-sectional	To determine the aetiology of non-Ebola admissions presenting with Ebola-like symptoms during the Ebola epidemic.	Tot: 278 (36 LF cases, 12.95% of 278 non-Ebola patients admitted with Ebola-like symptoms)	Of total 278 non-Ebola cases:<br>Mean Age 42.3 ± 8.6<br>Range (y) 2–75<br>Male 162 (58.27%)<br>Female 116 (41.73%)			Symptoms
Mahanty, S., Low levels of interleukin-8 and interferon-inducible protein-10 in serum are associated with fatal infections in acute Lassa fever. J Infect Dis. 2001. 183:1713-21	Cohort	To determine the levels of inflammatory cytokines and chemokines in patients with acute Lassa fever. To establish whether proinflammatory mediators derived from macrophages, endothelial cells, and other cellular sources are present during illness. To determine whether levels of proinflammatory mediators correlate with disease severity.	Total = 61, n=35 LF cases	Not reported			Association of levels of pro inflammatory mediators with disease severity
McCormick, J. B.,A case-control study of the clinical diagnosis and course of Lassa fever. J Infect Dis. 1987. 155:445-55	Cohort	To measure the frequency and case-fatality ratio of Lassa fever among febrile hospital admissions and to better delineate the clinical diagnosis and course of this disease.	Tot: 1087 febrile patients (441 patients with LF, 646 non-Lassa patients)	Patients > 10 years admitted<br>with a febrile illness to the medical ward			Signs and symptoms<br>Laboratory features<br>Incubation period<br>Case-fatality rate

McCormick, J. B.,Lassa fever. Effective therapy with ribavirin. N Engl J Med. 1986. 314:20-6	Non-randomized controlled study	To assess the therapeutic effectiveness of Ribavirin.	Tot: 312<br>n=63 Ribavirin intravenous (32/62 also received plasma),<br>n=39 Ribavirin oral,<br>n=53 Lassa convalescent plasma	Not reported	Ribavirin<br>Lassa fever convalescent plasma	Lassa convalescent plasma<br>No therapy	Mortality
Monath, T. P.A hospital epidemic of Lassa fever in Zorzor, Liberia, March-April 1972. Am J Trop Med Hyg. 1973. 22:773-9	Cross-sectional	Not reported		Not reported			Human-to-human nosocomial transmission
Monson, M. H., Endemic Lassa fever in Liberia. I. Clinical and epidemiological aspects at Curran Lutheran Hospital, Zorzor, Liberia. Trans R Soc Trop Med Hyg. 1984. 78:549-53	Cross-sectional	To assess clinical and epidemiological aspects of LF in the hospital.	Tot: 44 cases of LF	12 men, 32 women; 2 paediatric cases; 3 pregnant women<br>NB data reported inconsistent in the text and tables. These data from tables.			Mortality<br>Diagnostic accuracy<br>Leucocyte counts
Okokhere, P. O., Central nervous system manifestations of lassa fever in Nigeria and the effect on mortality. Journal of the Neurological Sciences. 2013. 333:e604	Cohort	To determine the CNS manifestations of LF and the effect on mortality.	Tot:162 patients	85 males, 77 females		Non-pathological respiratory symptoms.	Symptoms<br>Case fatality rate
Okokhere, P.,Pulmonary manifestation of lassa fever and the impact on mortality. European Respiratory Journal. Conference: European Respiratory Society Annual Congress. 2012. 40	Cohort	To determine the impact of pulmonary involvement on mortality in LF.	Tot: 65 LF patients	34 males, 31 females			Case fatality rate<br>Signs and symptoms
Panning, M., Laboratory diagnosis of Lassa fever, liberia. Emerg Infect Dis. 2010. 16:1041-3	Cross-sectional	Summarize our experiences from testing 184 patients with RT-PCR.	Tot:184 suspected cases of LF	Not reported	RT-PCR<br>Cell culture<br>Serology	Cell culture	Diagnostic methods
Price, M. E., A prospective study of maternal and fetal outcome in acute Lassa fever infection during pregnancy. BMJ. 1988. 297:584-7	Cohort	To compare the case fatality rate among pregnant women with Lassa fever with that among non-pregnant women of childbearing age who had been admitted to the hospital with Lassa fever.	Tot: 233 women (pregnant women: 68, non-pregnant women: 165)	Febrile women with pregnancy complications and non-pregnant women with Lassa fever	Ribavirin	Non-pregnant women with Lassa fever.	Case-fatality rate Drug therapy effect Human-to-human transmission Biochemical laboratory values
Roth, P. J.,Factors associated with mortality in febrile patients in a government referral hospital in the Kenema district of Sierra Leone. Am J Trop Med Hyg. 2015. 92:172-7	Cohort	To describe the clinical presentations, diagnoses, outcomes, and factors associated with increased mortality in febrile adult patients presenting to a government referral hospital in a Lassa-endemic region in rural Sierra Leone.	Tot: 21 patients with Lassa virus diagnosis	76% female, Median age 26 years (IQR 20-31)			Symptoms<br>Biochemical laboratory data<br>Mortality

Safronetz, D.,Annual Incidence of Lassa Virus Infection in Southern Mali. Am J Trop Med Hyg. 2017. 96:944-946	Cohort	To estimate annual incidence of LF by comparing population prevalence of seroconversion at two time points.	Tot: 1132<br>600 in 2015 (100%),<br>532 in 2016 (88.7%)	Average age 21 years (range 7 months–83 years) . 315 (52.5%) female, 285 (47.5%) male (Baseline data from Sogoba 2017)			Change in prevalence of seropositivity
Sogoba, N. Lassa Virus Seroprevalence in Sibirilia Commune, Bougouni District, Southern Mali. Emerg Infect Dis. 2016. 22:657-63	Cross-sectional	To estimate annual incidence of LF (linked to Safronetz, 2017).	Linked to Safronetz, 2017				
Trappier, S. G., Evaluation of the polymerase chain reaction for diagnosis of Lassa virus infection. Am J Trop Med Hyg. 1993. 49:214-21	Cohort	To evaluate polymerase chain reaction (PCR) and hybridization procedures for diagnosis of Lassa fever.	Tot: 98 specimens.<br>Tot: 195 patient sera	Not reported	PCR<br>Virus isolation	Gold standard: virus isolation	Sensitivity and specificity of PCR against virus isolation
U.S. Army Medical Research and Materiel Command; Valeant Pharmaceuticals International, Inc.. Treatment of Viral Hemorrhagic Fevers With Intravenous Ribavirin in Military Treatment Facilities. #journal#. 2009-2018.	Cohort - ongoing	To evaluate the safety of IV Ribavirin in subjects with a probable case of viral hemorrhagic fever (VHF) and to monitor the mortality of subjects with VHF who are treated with Ribavirin.<br>To evaluate IV Ribavirin drug intervention on clinical events that occur with VHF.	Estimated enrollment 50	Men and non-pregnant women, 17 - 65 years	Ribavirin		Adverse events Mortality Clinical events
U.S. Army Medical Research and Materiel Command; . Intravenous Ribavirin Protocol to Treat Individuals With Viral Hemorrhagic Fever. #journal#. 2016-2019.	Cohort - ongoing	Not reported	Estimated enrollment 30	Men and non-pregnant women, 18 - 65 years	Ribavirin		Adverse reactions
Winn, W. C., The pathology of human Lassa fever. Bull World Health Organ. 1975. 52:535-45	Cross-sectional	Reporting Pathology findings from reported and one unreported LF autopsy.	Tot:8	Unclear			Pathological findings
Zweighaft, R. M. Lassa fever: response to an imported case. N Engl J Med. 1977. 297:803-7	Cohort	Reporting response to imported LF case and identification, monitoring and outcomes of contacts.	Tot: 552 contacts on flights and community, hospitals n= 33 high risk exposures				Human-to-human nosocomial transmission

Abbreviations: Tot.: Total number, LF: Lassa fever, PEP: Post-exposure prophylaxis, IQR: Interquartile range, RT-PCR: Reverse transcriptase Polymerase chain reaction, SD: standard deviation
